# Dual membrane-spanning anti-sigma 2 controls OMV biogenesis and colonization fitness in *Bacteroides thetaiotaomicron*

**DOI:** 10.1128/jb.00442-25

**Published:** 2026-03-05

**Authors:** Evan J. Pardue, Tengfei Zhong, Nichollas E. Scott, Biswanath Jana, Wandy Beatty, Juan C. Ortiz-Marquez, Mohammed Kaplan, Clay Jackson-Litteken, Mario F. Feldman

**Affiliations:** 1 Department of Molecular Microbiology, Washington University School of Medicine 12275 , St. Louis, Missouri, USA; 2 Department of Microbiology, University of Chicago 456539, Chicago, Illinois, USA; 3 Department of Microbiology and Immunology, The Peter Doherty Institute for Infection and Immunity, University of Melbourne 198084, Parkville, Victoria, Australia; 4 Biology Department, Boston College 6019, Chestnut Hill, Massachusetts, USA; 5 Department of Microbiology and Immunology, University of Arkansas for Medical Sciences 12215, Little Rock, Arkansas, USA; University of Southern California, Los Angeles, California, USA

**Keywords:** vesicle, gut, PULs, regulation, OMVs, bacteroides, sigma factors

## Abstract

*Bacteroides spp*. are gram-negative, gut commensals that shape the enteric landscape by producing outer membrane vesicles (OMVs) that degrade dietary fibers and traffic immunomodulatory biomolecules. Understanding the mechanism behind OMV biogenesis in *Bacteroides spp*. is necessary to determine their role in the gut. Recent studies showed that mutation of dual membrane-spanning anti-sigma factor 1 (Dma1) increased OMV production in *Bacteroides thetaiotaomicron* (*Bt*) by modulating the expression of its downstream regulon. Additional members of the Dma family have been identified, but very little is known regarding their roles in *Bt*. Here, we investigate the role of Dma2 in controlling OMV biogenesis in *Bt*. We employ biochemical and proteomic analyses to show that mutation of *dma2* increases OMV production. This induction is dependent on the expression of its cognate sigma factor, *das2*, but the precise mechanism by which *dma2* increases OMV biogenesis remains elusive. Transcriptome analyses revealed that *Δdma2* displays decreased expression of select polysaccharide utilization loci (PULs) that primarily target host-associated glycans. Follow-up comparative proteomics showed that the PUL repertoire was most impacted in the OMV fraction. *In vitro* growth assessments confirmed that *Δdma2* exhibits delayed growth in the presence of select host-associated glycans. *In vivo* co-colonization studies in mice revealed that *Δdma2* is outcompeted by the wild-type in the gut, which indicates that Dma2 is a key determinant of colonization fitness in *Bt*. Altogether, these findings expand our knowledge of the Dma family’s role in OMV biogenesis and demonstrate their importance in *Bacteroides* physiology.

**IMPORTANCE:**

Dual membrane-spanning anti-sigma factors (Dma) are a novel class of regulatory proteins found solely among Bacteroidota. Previous studies demonstrated the importance of Dma1 in vesiculation, but the overall role of the Dma family in Bacteroides physiology remains poorly understood. Here, we show that Dma2 modulates vesiculation and the expression of select polysaccharide utilization loci (PULs) that target host-associated glycans *in vitro*. Mouse studies revealed that Dma2 is an important fitness determinant *in vivo* when competing against kin bacteria. This work begins characterizing the multifaceted involvement of Dma2 in OMV biogenesis, PUL regulation, and colonization fitness.

## INTRODUCTION

The gut microbiota is the consortium of trillions (~10^12^) of microbes that inhabit the human gastrointestinal tract (1–3). This collection of microbes promotes the proper development of the gut epithelium and immune system through various mechanisms (2). *Bacteroides spp*. are one of the most abundant genera, making up ~40% of the bacterial species in the human gut. These microbes help maintain intestinal homeostasis by outcompeting select pathogens, breaking down indigestible dietary fibers, producing short-chain fatty acids, and modulating intestinal immunity to reduce inflammation (3–6).

*Bacteroides spp*. can stably colonize and successfully compete within the gut due to their ability to utilize a diverse array of dietary polysaccharides and host-associated glycans to promote their growth (3, 4). This process is mediated by numerous encoded polysaccharide utilization loci (PULs) that can account for ~20% of the genome in *Bacteroides spp*. PULs are complex nutrient acquisition systems that sense, degrade, and import polysaccharides and other nutrients to be utilized by *Bacteroides spp*. (4, 6–8). Each PUL targets a particular class of polysaccharide and is characterized by the presence of SusC and SusD orthologs. SusD-like proteins are surface-exposed lipoproteins that bind to polysaccharides, which enables them to be broken down further by surface glycosyl hydrolases and imported into the periplasm via a SusC-like TonB-dependent outer membrane (OM) porin (6, 9)

Previous mass spectrometry (MS) analyses revealed that outer membrane vesicles (OMVs) from *Bacteroides thetaiotaomicron* (*Bt*) and *Bacteroides fragilis* (*Bf*) are preferentially enriched with surface-exposed glycosyl hydrolases, SusD-like proteins, and other proteins typically encoded in PULs (10–12). OMVs are small, spherical, membranous compartments derived from the active blebbing of the OM of Gram-negative bacteria to traffic cellular contents (13). Due to their glycolytic activity, *Bacteroides* OMVs are viewed as “public goods” because they can degrade various intestinal fibers at a distance, and the resulting breakdown products are readily accessible to kin bacteria and other commensal microbes (11, 14, 15). Producing OMVs as “public goods” is energetically costly; hence, *Bacteroides spp*. must tightly control the co-expression of PULs along with OMV biogenesis (11, 13). We recently reported evidence for this model, demonstrating that *Bt* alters their OMV PUL repertoire to adapt to the extracellular glycan landscape (11). This phenomenon provides further support for the idea that OMVs produced by *Bacteroides spp.* are important for these microbes to effectively compete in the gut. Despite their importance, very little is known regarding how OMV biogenesis and regulation occurs. Determining how *Bacteroides* OMVs are produced and regulated is key to understanding the physiology of these microbes and how they function in the gut.

Our recent studies have gained insight into how OMV biogenesis is regulated in *Bt* (11, 16). Briefly, we expressed fluorescent OMV reporters in live cells and employed fluorescence microscopy to visualize OMVs actively blebbing from the OM of live *Bt* cells (11). By adapting this visualization system, we developed an OMV reporter screen to allow the identification of genes involved in OMV biogenesis and regulation *in vitro* in a high-throughput manner (16). We found that mutation of Dual Membrane-spanning Anti-sigma factor 1 (*Δdma1*) induces OMV production in *Bt* by relieving the repression on its cognate ECF21 family sigma factor, *das1* (16). Dma1 is the first representative of a new class of structurally novel anti-sigma factors that have domains spanning from the OM into the cytosol (16). Additional Dma family members, termed Dma2 and Dma3, were identified in *Bt* (16). In this work, we investigate the role of Dma2 in *Bt*. Our findings demonstrate that Dma2 plays dual roles in modulating OMV biogenesis and is an important determinant of colonization fitness by regulating host-glycan targeting PULs.

## RESULTS

### Mutation of Dma2 (BT_1558) increases OMV production

**Fig 1:**
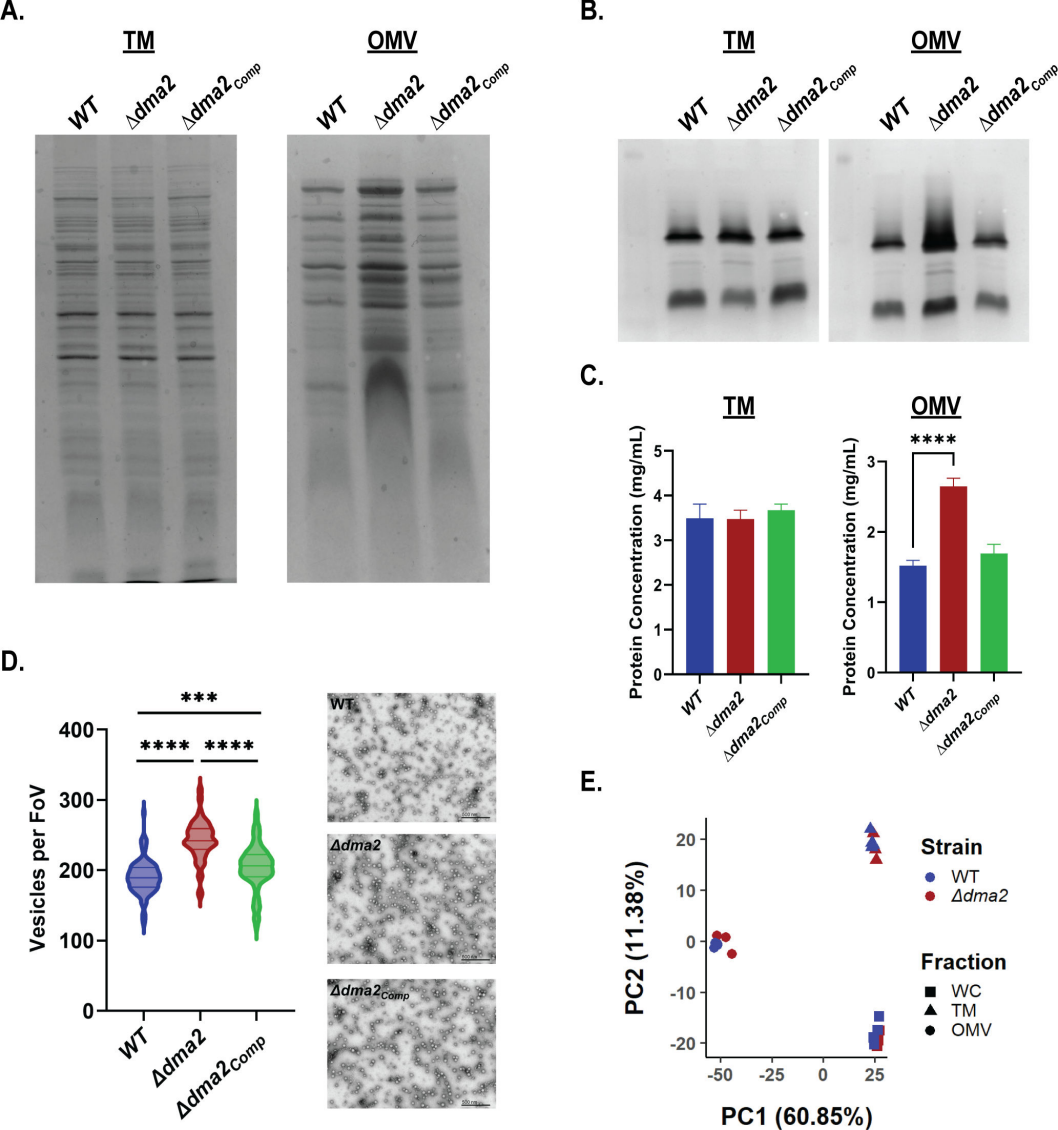
Mutation of Dma2 leads to increased OMV biogenesis in *Bt*. (**A**) Coomassie Blue stain comparing electrophoretic profiles between TM and OMV fractions from *Bt* WT, Δ*dma2*, and Δ*dma2_Comp_*. Samples were normalized by OD_600_ prior to being run on 10% SDS-PAGE gels. This suggests that deletion of *dma2* induces vesiculation in *Bt*. (**B**) LPS Silver Stains and (**C**) Bio-Rad DC protein assays comparing TM and OMV fractions from *Bt* WT, Δ*dma2*, and Δ*dma2_Comp_*. These show that Δ*dma2* contains more LPS and proteins in its OMV fraction, which is consistent with increased OMV production. Data represents the mean and standard error of three biological replicates performed in triplicate. (**D**) TEM confirms that *Δdma2* produces significantly more OMVs than the WT. Left: Results of the quantification of 90 TEM images of OMVs from the OMV fraction from each strain (FoV: Field of view). Right: Representative TEM images of OMVs from each strain. Three biological replicates of aliquots from the OMV fraction of each strain were fixed onto grids in triplicate (in Materials and Methods). Ten random images were taken from each grid (*n* = 90 per strain), and OMVs were counted manually. (**E**) Principal component analysis (PCA) of WC, TM, and OMV proteomic data from *Bt* WT and Δ*dma2*. This demonstrates that the overall composition of each cellular fraction is similar between the two strains. For panels C and D, two-tailed unpaired *t*-tests were performed to determine statistical significance. Significance threshold corresponds to: (*) *P*-value ≤ 0.05, (**) *P*-value ≤ 0.01, (***) *P*-value ≤ 0.001, and (****) *P*-value ≤ 0.0001.

Our preliminary experiments suggested that Dma2 controls OMV biogenesis in a similar manner to Dma1 in *Bt* (16). To begin our analysis, we isolated total membranes (inner and outer membranes; TM) and OMVs from the wild-type (WT), *Δdma2*, and its corresponding complemented strain (*Δdma2_Comp_*) and analyzed their protein profiles via SDS-PAGE, followed by Coomassie staining. We found that *Δdma2* exhibited a distorted electrophoretic profile when compared to the WT and *Δdma2_Comp_* (Fig. 1A). The OMV fractions obtained from hypervesiculating strains display irregular SDS-PAGE profiles, due to the increased abundance of lipopolysaccharide (LPS), a key OMV structural component, in these samples (16). Furthermore, we performed LPS Silver Stains to measure the relative amounts of LPS and quantified the total protein present in the TM and OMV fractions from the WT, *Δdma2*, and *Δdma2_Comp_* (Fig. 1B and C). Although no differences were observed in the content of LPS and proteins in the TM fractions, the OMV fraction from *Δdma2* contained significantly more LPS and protein when compared to the WT and *Δdma2_Comp_* (Fig. 1B and C). Together, these findings all suggest that mutation of *dma2* causes *Bt* to hypervesiculate. To directly quantify OMV production, we isolated OMVs from the WT, *Δdma2,* and *Δdma2_Comp_* strains, imaged them by transmission electron microscopy (TEM), and then quantified the number of vesicles present per image. Quantification of OMVs by TEM confirmed that *Δdma2* increases OMV production by ~50% when compared to the WT (Fig. 1D). The complemented strain was found to be significantly different from the WT, but this is likely due to partial complementation. Our findings indicate that the observed phenotypes are due to increased vesiculation and not compositional changes in the contents of the OMV fraction in *Δdma2* (Fig. 1D).

Previous studies have demonstrated that *Bacteroides* OMVs contain select protein cargo that consists primarily of surface-exposed lipoproteins derived from PULs (10–12). To ensure that the increase in vesiculation in *Δdma2* is not caused by cell lysis, we performed comparative proteomic analyses of whole cells (WC), TM, and OMV from the WT and *Δdma2*. The resulting PCA shows that each fraction from the WT and *Δdma2* contains similar protein composition (Fig. 1E). Vesicles generated by lysis usually carry ribosomal proteins and other cytoplasmic components. However, since the proper OMV cargo selection is maintained in *Δdma2*, we can rule out that the increased OMV production observed in *Δdma2* is due to membrane instability and cell lysis.

**Fig 2:**
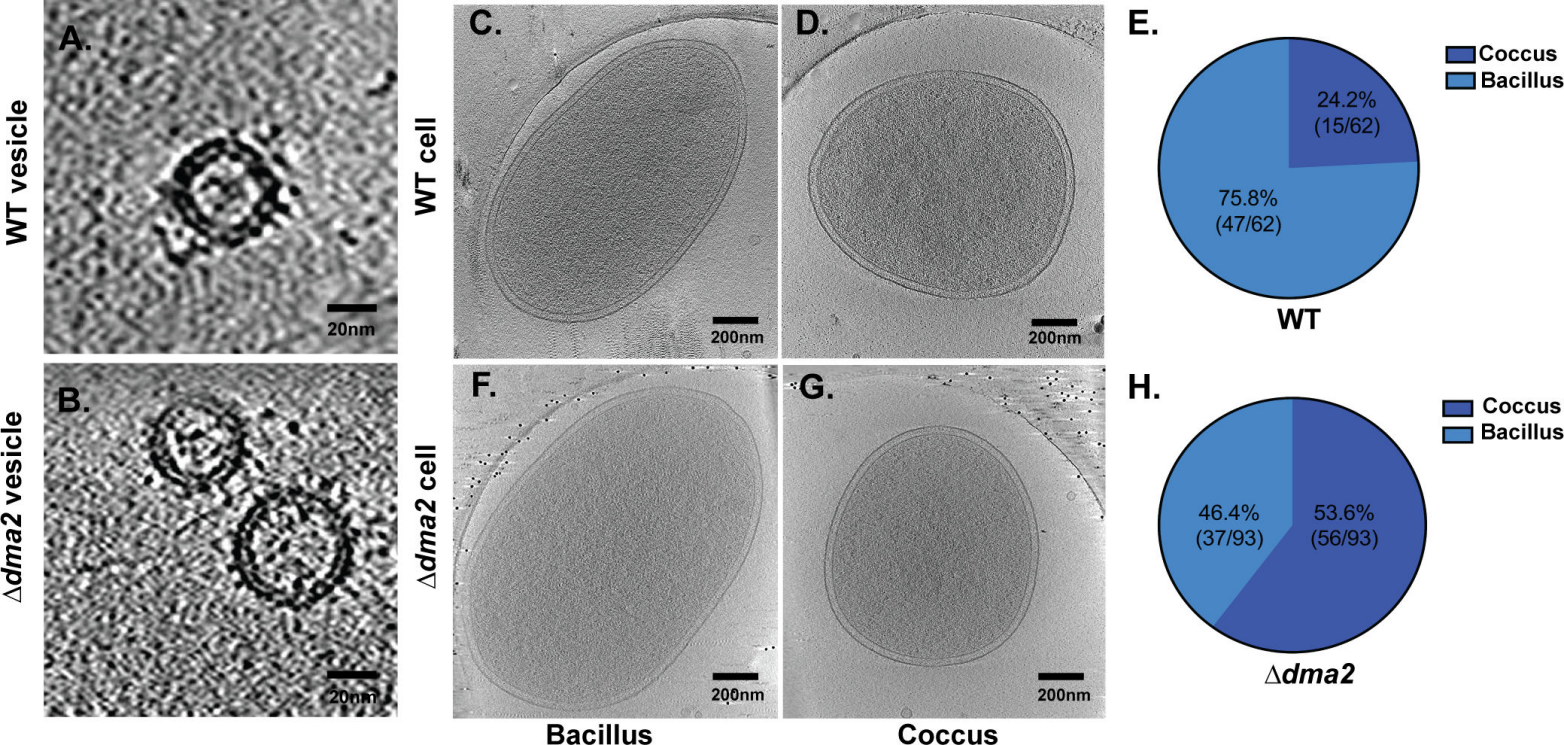
Mutation of Dma2 causes morphological changes in *Bt* cells. Representative slices through cryo-electron tomograms of OMVs from (**A**) *Bt* WT and (**B**) *Δdma2*. Slices through cryo-electron tomograms of rod-shaped and coccoid cells, and their corresponding ratios from (**C-E**) *Bt* WT and (**F-H**) *Δdma2*. This shows that the mutation of *dma2* increases the number of coccoid cells present in *Bt*.

Next*,* we performed cryo-electron tomography (cryoET) on the WT and *Δdma2* to assess the morphology of *Bt* OMVs. The size and electron density of OMVs were not impacted in *Δdma2* (Fig. 2A and B; Fig. S1). However, we found that *Δdma2* exhibited a higher proportion of coccoid cells (53.6%) when compared to the WT (24.2%) (Fig. 2C through H). This suggests a potential link between OMV production and cell shape maintenance.

### Das2 (BT_1559) is required to induce OMV production in *Δdma2*

**Fig 3:**
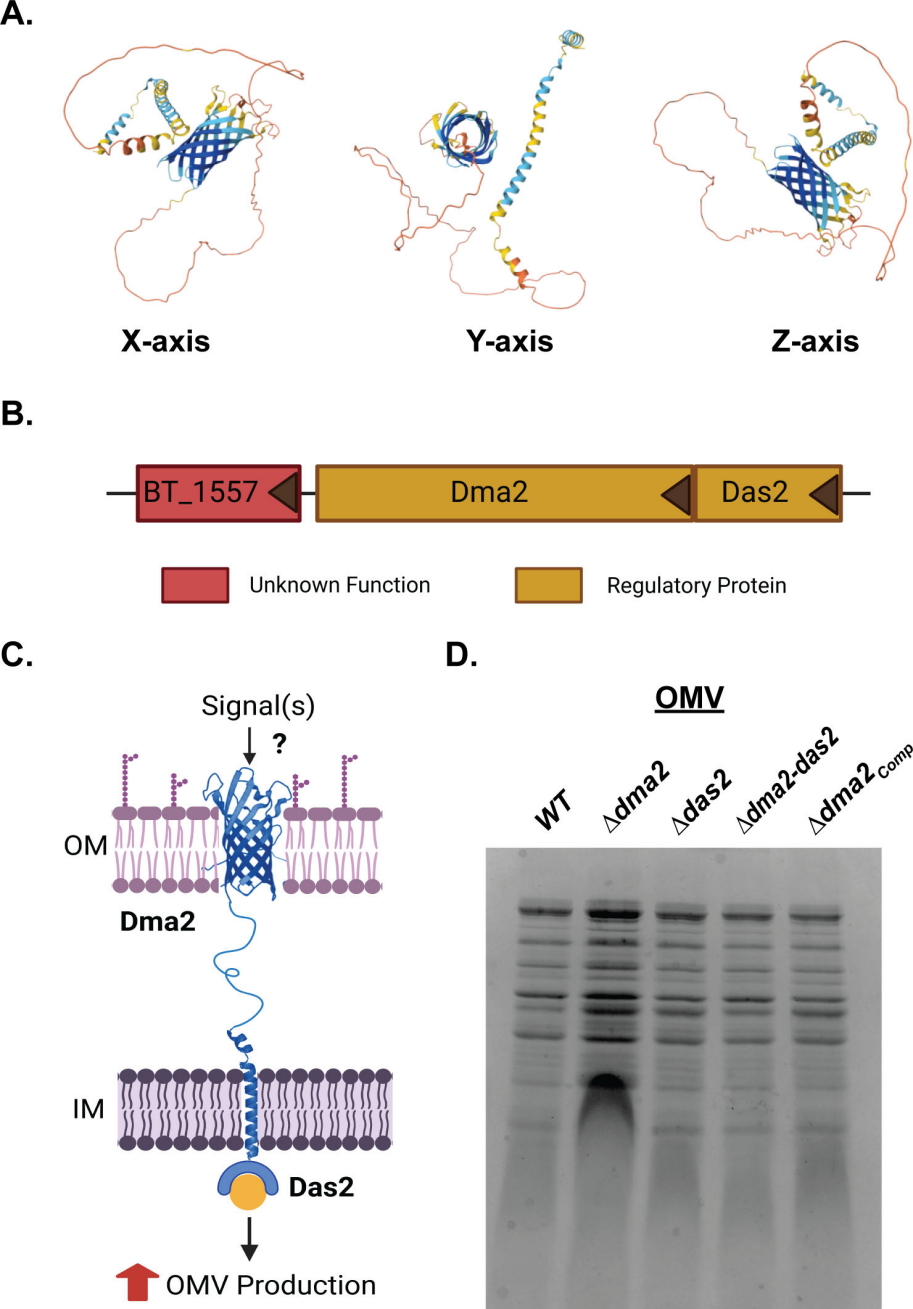
ECF21 family sigma factor, *das2,* is required to induce OMV biogenesis in *Bt*. (**A**) AlphaFold structural predictions of Dma2 (19). (**B**) Schematic and putative functions of each gene present in the *dma2* operon (Created with BioRender.com). (**C**) Proposed model of how Dma2 induces OMV production in *Bt* by controlling the activity of Das2 (Created with BioRender.com). (**D**) Coomassie Blue stain comparing electrophoretic profiles of OMV fractions from *Bt* WT, *Δdma2*, *Δdas2, Δdas2-dma2*, and *Δdma2_Comp_*. Samples were normalized by OD_600_ values and run on 10% SDS-PAGE gel. This confirms that deletion of *das2* in the *Δdma2* background restores WT levels of vesiculation.

Dma2, like other Dma family members, exhibits a unique domain organization, consisting of (i) an N-terminal anti-sigma binding domain, (ii) a transmembrane helix, (iii) a long, intrinsically disordered tether-like region, and (iv) a C-terminal β-barrel domain (Fig. 3A). We showed previously that Dma1 modulates OMV biogenesis by directly controlling the activity of its cognate sigma factor, Das1 (16). Dma2 is encoded in a three-gene operon with BT_1557, a protein of unknown function, and BT_1559 (*das2*), a putative ECF21 family sigma factor (Fig. 3B). ECF21 family sigma factors are found solely amongst Bacteroidota and are encoded adjacent to Dma family members (16–18). This strongly suggests that Dma2 and Das2 form a sigma/anti-sigma pair (Fig. 3C). We hypothesized that the hypervesiculation observed in *Δdma2* is due to the liberation and subsequent activation of Das2. To confirm whether Das2 is required to induce OMV biogenesis in *Bt*, we generated clean *das2* deletion mutants in the WT (*Δdas2*) and *Δdma2* background (*Δdma2-das2*). Growth curves were performed with these strains and revealed that *Δdma2* grew slightly slower than the WT and other tested strains in BHI media (Fig. S2). Next, we isolated OMVs from the WT, *Δdma2, Δdas2, Δdma2-das2,* and *Δdma2_Comp_* and compared the electrophoretic profiles, as a proxy for OMV biogenesis, by SDS-PAGE, followed by Coomassie staining. While we observed no phenotype in *Δdas2*, we found that *Δdma2-das2* displayed a WT electrophoretic profile (Fig. 3D). This confirms that the increased OMV production observed in *Δdma2* requires the activity of its sigma factor, *das2*.

### Dma2 controls OMV biogenesis in a manner that is distinct from that of Dma1

**Fig 4:**
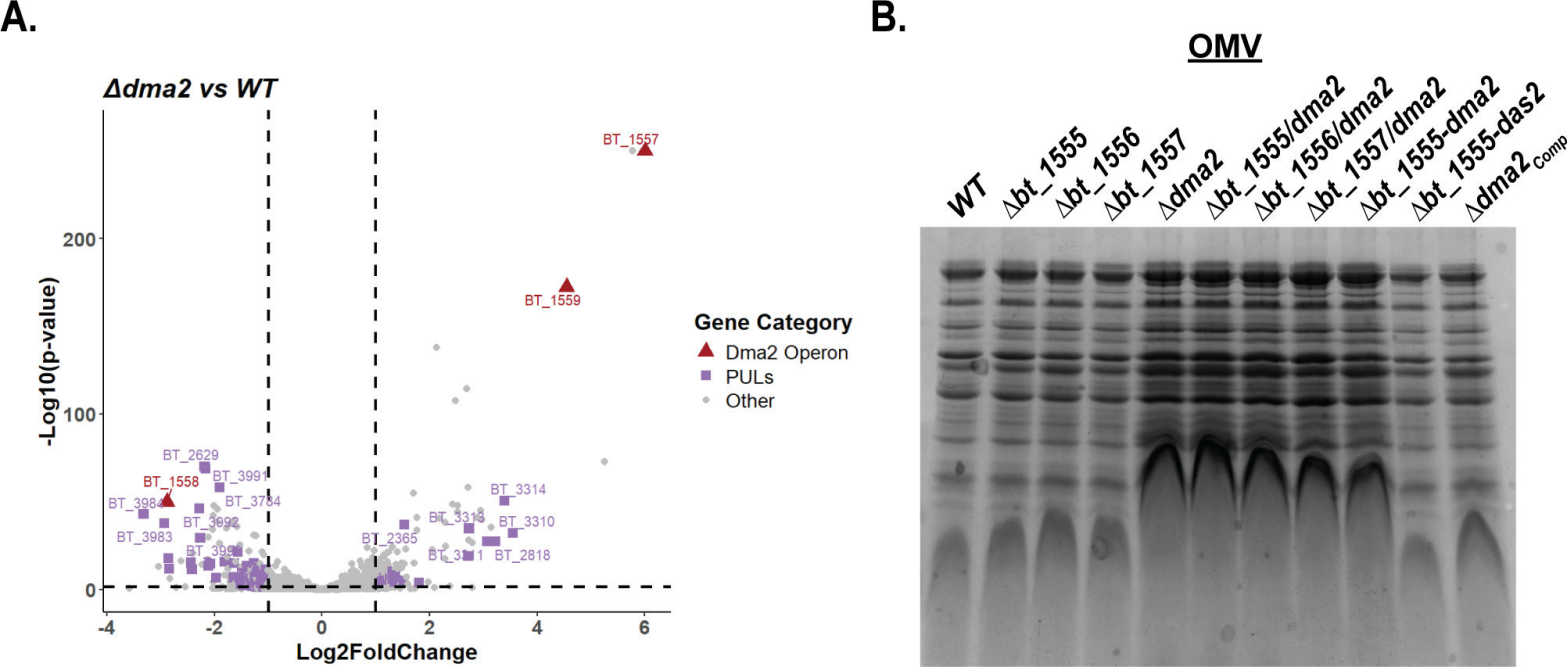
Dma2 primarily regulates its own operon and PULs targeting host-associated glycans in *Bt*. (**A**) Volcano plot representations of transcriptome data comparing *Bt* WT and *Δdma2* (Data sets S1 and S2). (**B**) Coomassie Blue stain of OMV fractions isolated from *Bt* WT and strains containing deletions in the downstream genes of the *dma2* operon. Samples were normalized by OD_600_ and run on 10% SDS-PAGE. This experiment shows that the *dma2* operon downstream genes are not responsible for the induction of OMV production observed in *Δdma2*. In the gel, *Δbt_1555/dma2* represents the *bt_1555* and *dma2* double mutant, *Δbt_1556/dma2* represents the *bt_1556* and *dma2* double mutant, *Δbt_1557/dma2* represents the *bt_1557* and *dma2* double mutant, *Δbt_1555-dma2* is lacking *bt_1555*, *bt_1556*, *bt_1557*, and *dma2*, while *Δbt_1555-das2* is missing *bt_1555*, *bt_1556*, *bt_1557*, *dma2*, and *das2*.

To investigate how Dma2 controls vesiculation, we compared the transcriptome of *Δdma2* to that of the WT. Our analysis revealed that the most differentially regulated genes belonged to two main categories: (i) genes encoded in and adjacent to the *dma2* operon and (ii) genes that are components of PULs (Fig. 4A; Data sets S1 and S2).

In *Δdma2, bt_1555* (Log_2_FC: 2.48), *bt_1556* (Log_2_FC: 5.78), *bt_1557* (Log_2_FC: 6.00), and *das2* (*bt_1559*) (Log2FC: 4.54) were among the most upregulated genes (Fig. 3B; Fig. 4A; Table S2; Data set S1). No studies have attributed functions to these genes; however, Foldseek predicts that BT_1555 is structurally similar to enoyl-acyl carrier protein (ACP) reductases and nitronate monooxygenases, while BT_1556 and BT_1557 are annotated as DUF4858- and DUF4943 domain-containing proteins, respectively (20). To test whether these genes impact OMV production, we generated mutant strains in each of these genes in the *Δdma2* background. OMV analysis revealed that deletion of the upregulated genes encoded near Dma2 and Das2 does not have an impact on OMV biogenesis (Fig. 4B).

We previously showed that *Δdma1* induces the expression of *nigD1* (*bt_4005*) along with other select genes in *Bt* (16). NigD1 belongs to a class of proteins called NigD-like proteins that are found solely amongst Bacteroidota, and it is required to increase OMV production in *Δdma1* (16). Since Dma1 and Dma2 both belong to the same family and increase OMV biogenesis, we hypothesized that mutation of *dma2* could also induce OMV production by increasing the expression of *nigD1*. However, our RNA-seq analysis revealed that the expression of *nigD1* (Log_2_FC: −0.79) was slightly downregulated in Δ*dma2*, which counters this idea (Table S2; Data set S1).

Finally, to determine whether there are shared genes regulated by both *dma1* and *dma2* that could provide additional insight regarding how they modulate OMV biogenesis, we compared our previous transcriptome data from *Δdma1* (16) to that collected from *Δdma2*. We found that genes encoding a putative type V pilus (*bt_2655-2660*) (21), an orphan ECF type sigma factor (*bt_2569*), the *dma3* locus (*bt_2778-2779*) (16), and many hypothetical proteins are upregulated, while components from PUL36 (α-mannan/host N-glycans), PUL52 (unknown), PUL67 (mucin-O-glycans), PUL68 (α-mannan/host N-glycans) (22, 23), S-layer proteins (*bt_1926-1927*) (24), and glycine betaine/L-proline transport system permeases (*bt_1750-1751*) (25) are downregulated in both *Δdma1* and *Δdma2* (Table S3; Data sets S3 and S4). Many of the shared genes identified here are not likely to be implicated in OMV biogenesis, but the *dma3* locus is of significant interest. Since the *dma3* locus is upregulated in both strains, this suggests that there is potentially crosstalk occurring between members of the Dma family. To determine whether *dma3* plays a role in inducing OMV biogenesis, we generated clean deletion mutants in *dma3* in the WT, *Δdma1*, and *Δdma2* backgrounds prior to isolating the OMV fraction and visualizing the electrophoretic profile by SDS-PAGE followed by Coomassie staining. However, mutation of *dma3* does not revert the distortion in the electrophoretic profiles observed in *Δdma1* and *Δdma2*. This indicates that *dma3* activity is not required for these strains to hypervesiculate (Fig. S3). Additional studies are required to determine the role of Dma3 in *Bt*. Altogether, our findings support the conclusion that *Δdma1* and *Δdma2* induce OMV production through distinct regulatory cascades.

### Absence of Dma2 results in altered OMV cargo selection

PULs are complex, nutrient acquisition systems encoded by Bacteroidota that enable them to utilize a wide array of dietary-, microbial-, and host-derived glycans (4, 7, 8, 23). The transcriptomic data from *Δdma2* revealed that genes in select PULs are differentially regulated. We found that PUL36 (α-mannan/host N-glycans), PUL52 (unknown substrate), PUL68 (α-mannan/host N-glycans), and PUL72 (mucin-O-glycans/high mannose mammalian N-glycan) are the most repressed PULs when compared to the WT. On the other hand, PUL38 (mucin-O-glycans) and PUL56 (1,6-β-glucan) were induced in *Δdma2* when compared to the WT (Fig. 4A; Table 1; Data sets S1 and S2).

**TABLE 1:** List of differentially expressed PUL genes from *Δdma2* vs *WT* RNA sequencing

Downregulated PULs					
PUL	Gene (new locus tag)	Gene (old locus tag)	Log2FC	*P*adj	Substrate
PUL68	BT_RS19095	BT_3787	−2.8421101	6.585E-11	α-Mannan/host N-glycans
	BT_RS19100	BT_3788	−2.4206965	8.656E-11	α-Mannan/host N-glycans
	BT_RS19060	BT_3779	−2.0816286	8.501E-14	α-Mannan/host N-glycans
	BT_RS19105	BT_3789	−1.972928	3.105E-06	α-Mannan/host N-glycans
	BT_RS19085	BT_3784	−1.905731	3.898E-56	α-Mannan/host N-glycans
	BT_RS19110	BT_3790	−1.5110252	0.0091832	α-Mannan/host N-glycans
	BT_RS19065	BT_3s780	−1.4576049	0.0204024	α-Mannan/host N-glycans
	BT_RS19120	BT_3792	−1.4516525	1.902E-08	α-Mannan/host N-glycans
	BT_RS19115	BT_3791	−1.4283173	0.0010753	α-Mannan/host N-glycans
	BT_RS19090	BT_3786	−1.3291173	1.298E-05	α-Mannan/host N-glycans
	BT_RS19045	BT_3776	−1.3034711	0.0621142	α-Mannan/host N-glycans
	BT_RS19050	BT_3777	−1.2124131	0.0627374	α-Mannan/host N-glycans
	BT_RS19070	BT_3781	−1.1770001	3.468E-10	α-Mannan/host N-glycans
PUL52	BT_RS16400	BT_3239	−1.8079871	1.037E-14	Unknown
	BT_RS16395	BT_3238	−1.6402074	2.635E-06	Unknown
	BT_RS16415	BT_3240	−1.5664591	1.853E-20	Unknown
	BT_RS16390	BT_3237	−1.4894948	1.561E-08	Unknown
	BT_RS16385	BT_3236	−1.4012068	1.418E-12	Unknown
	BT_RS16380	BT_3235	−1.3593774	1.576E-20	Unknown
	BT_RS16425	BT_3242	−1.2688034	4.602E-14	Unknown
	BT_RS16420	BT_3241	−1.252067	1.2E-06	Unknown
	BT_RS16430	BT_3243	−1.2089576	5.266E-09	Unknown
	BT_RS16435	BT_3244	−1.0403248	1.594E-10	Unknown
PUL72	BT_RS20105	BT_3984	−3.3194878	2.338E-41	Mucin-O-glycans/high mannose mammalian N-glycan
	BT_RS20100	BT_3983	−2.9338803	2.737E-36	Mucin-O-glycans/high mannose mammalian N-glycan
	BT_RS20110	BT_3985	−2.8586692	8.722E-17	Mucin-O-glycans/high mannose mammalian N-glycan
	BT_RS20115	BT_3986	−2.4411336	3.071E-14	Mucin-O-glycans/high mannose mammalian N-glycan
	BT_RS20145	BT_3992	−2.2819004	2.178E-44	Mucin-O-glycans/high mannose mammalian N-glycan
	BT_RS20135	BT_3990	−2.2630706	3.503E-28	Mucin-O-glycans/high mannose mammalian N-glycan
	BT_RS20140	BT_3991	−2.1626853	9.057E-67	Mucin-O-glycans/high mannose mammalian N-glycan
	BT_RS20120	BT_3987	−2.1192517	1.675E-12	Mucin-O-glycans/high mannose mammalian N-glycan
	BT_RS20125	BT_3988	−1.5356378	5.419E-06	Mucin-O-glycans/high mannose mammalian N-glycan
PUL36	BT_RS13295	BT_2629	−2.1856439	3.453E-68	α-Mannan/host N-glycans
	BT_RS13290	BT_2628	−1.6324335	5.416E-14	α-Mannan/host N-glycans
	BT_RS13280	BT_2626	−1.1388348	2.477E-06	α-Mannan/host N-glycans
	BT_RS13260	BT_2622	−1.126785	2.413E-09	α-Mannan/host N-glycans
	BT_RS13270	BT_2624	−1.0544695	1.269E-06	α-Mannan/host N-glycans
PUL77	BT_RS21005	BT_4163	−1.2380494	1.52E-06	Unknown
	BT_RS21010	BT_4164	−1.138667	0.000873	Unknown
	BT_RS20950	BT_4152	−1.1385313	0.0020674	Unknown
	BT_RS21015	BT_4165	−1.037398	0.0047151	Unknown
PUL74	BT_RS20620	BT_4085	−1.3791482	4.887E-05	Host glycans (unknown type, PMG phase 2)
	BT_RS20585	BT_4078	−1.0984951	0.129151	Host glycans (unknown type, PMG phase 2)
PUL67	BT_RS18915	BT_3750	−1.1872617	2.098E-11	Mucin-O-glycans
	BT_RS18910	BT_3749	−1.082215	6.278E-06	Mucin-O-glycans
PUL5	BT_RS01345	BT_0273	−1.1012221	0.029263	Unknown
	BT_RS01330	BT_0270	−1.0807573		Unknown
PUL80	BT_RS21705	BT_4299	−1.3966409	1.584E-06	Host glycans (unknown type, likely mucin O-glycans)
PUL7	BT_RS01770	BT_0363	−1.129689		Host/residual dietary glycans (unknown type)
PUL83	BT_RS22555	BT_4472	−1.0642391	0.0007893	Unknown
PUL73	BT_RS20385	BT_4039	−1.0457806	0.000107	Mucin-O-glycans
PUL75	BT_RS20765	BT_4114	−1.0406832	0.0011298	Host/residual dietary glycans (unknown type)
PUL71	BT_RS19970	BT_3958	−1.0170786	7.251E-06	Mucin O-glycans (core 1 disaccharide)

Upregulated PULs					
PUL	Gene (new locus tag)	Gene (old locus tag)	Log2FC	*P*adj	Substrate
PUL56	BT_RS16765	BT_3310	3.54932514	7.612E-31	1,6-β-Glucan
	BT_RS16785	BT_3314	3.3944726	8.14E-49	1,6-β-Glucan
	BT_RS16770	BT_3311	3.06987465	5.44E-26	1,6-β-Glucan
	BT_RS16780	BT_3313	2.73209628	2.017E-33	1,6-β-Glucan
	BT_RS16775	BT_3312	2.71127794	7.675E-18	1,6-β-Glucan
PUL38	BT_RS14290	BT_2818	3.22299809	5.238E-26	Mucin O-glycans
	BT_RS14295	BT_2819	1.79993412	0.0012312	Mucin O-glycans
	BT_RS14305	BT_2821	1.51203928	0.0001649	Mucin O-glycans
	BT_RS14310	BT_2822	1.3794696	0.0005488	Mucin O-glycans
	BT_RS14300	BT_2820	1.30517093	1.698E-05	Mucin O-glycans
PUL14	BT_RS05220	BT_1038	1.34356584	0.0006861	Mucin O-glycans
	BT_RS05210	BT_1036	1.15233709	3.2E-06	Mucin O-glycans
	BT_RS05215	BT_1037	1.11853749	0.0002727	Mucin O-glycans
PUL31	BT_RS11950	BT_2365	1.53114035	1.388E-35	Unknown
PUL66	BT_RS18675	BT_3704	1.18356674	0.0001789	Starch

**Fig 5:**
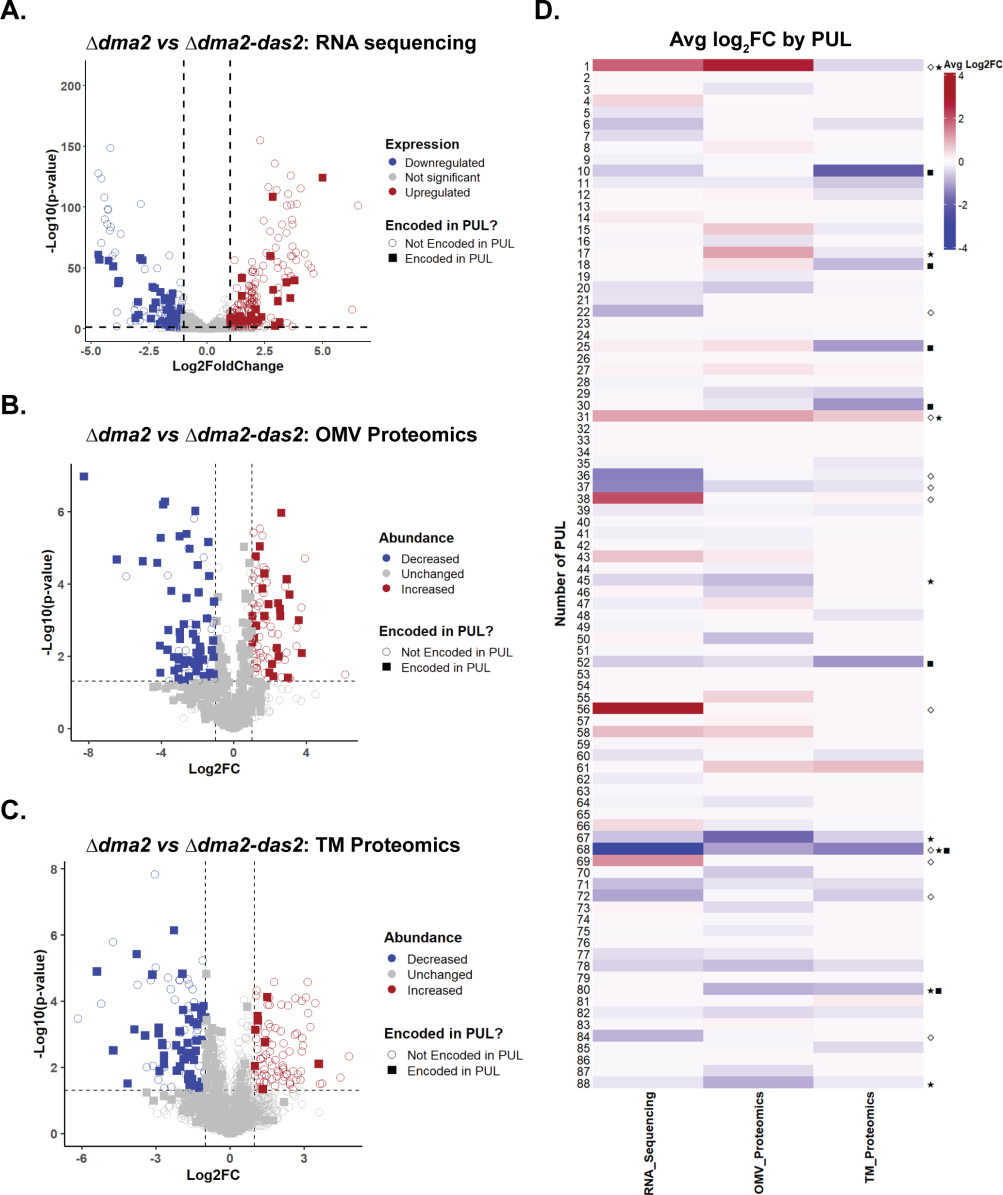
Select PULs targeting host-associated glycans are downregulated in *Δdma2*. Volcano plot representations of (**A**) RNA-seq, (**B**) OMV, and (**C**) TM proteomic data comparing *Bt Δdma2* and *Δdma2-das2* (Data sets S5 through S7). These analyses reveal that *Δdma2* primarily alters PULs that target host-associated glycans. These changes are most prevalent in the OMV fraction. (**D**) Heatmap comparing the differential expression (Average log2FC) of each PUL present in *Bt* from *Bt Δdma2* and *Δdma2-das2* transcriptome and proteome data sets. Average log2FC was calculated by filtering out each gene found to be encoded within a particular PUL and averaging the significant log2FC values (log2FC ≥ |±1| and *P*-value < 0.05). Non-significant log2FC values were included when calculating the Average log2FC for each PUL, but these were assigned the arbitrary value of 0. To ascribe significance, we used the cutoff |Average Log2FC| > 0.8. These analyses enabled us to distinguish whether entire PULs are altered in *Δdma2* or if only specific components of certain PULs are changed. PULs that are significantly altered are indicated by the following symbols: “◊” for RNA sequencing, “■” for TM proteomics, and “★” for OMV proteomics.

Since Dma2 is an anti-sigma factor that functions by controlling the activity of Das2, we propose that the selective dysregulation of PULs observed in *Δdma2* is caused by Das2 activity. Since the WT bacteria express basal levels of Das2, to gain more insight regarding how Dma2 and Das2 modulate the expression of PULs in *Bt*, we conducted additional transcriptomic analyses that compared *Δdma2*, where Das2 is constitutively active, to *Δdma2-das2*, where the system is completely inactive. Like the previous RNA-seq, the *dma2* operon and its neighboring genes were the most induced, while the expression of various PULs was also changed (Table S4; Data set S5). In this new data set, additional genes related to capsule biosynthesis were also differentially regulated, which we postulate is a consequence of phase variation, a common phenomenon in Bacteroides (Data set S5) (26, 27). Remarkably, PUL1 (unknown), PUL31 (unknown), PUL38 (mucin-O-glycans), PUL56 (1,6-β-glucan), and PUL69 (α-mannan/host N-glycans) were significantly induced, while PUL22 (levan/fructooligosaccharides), PUL36 (α-mannan/host N-glycans), PUL37 (ribose/ribonucleosides), PUL68 (α-mannan/host N-glycans), PUL72 (mucin-O-glycans), and PUL84 (mucin-O-glycans) were significantly repressed in *Δdma2* (Fig. 5A; Table S5; Data set S5). Overall, a majority of the differentially expressed PUL genes identified (44/68; ~65%) are predicted to target host glycans (23).

To evaluate whether the modulation of PUL expression observed in *Δdma2* affects OMV cargo, we performed comparative proteomic analyses on OMVs isolated from *Δdma2* and *Δdma2-das2*. In our analysis, we also included total membranes (inner and outer; TM). In *Δdma2* OMVs, PULs comprised ~50% of the total proteins found to be significantly altered. On the contrary, the TM fraction displayed fewer changes to PULs (Fig. S4; Data sets S6 and S7). Our analyses revealed that PULs are primarily repressed at the protein level in these subcellular fractions of *Δdma2*, while very few are induced (Fig. 5B and C; Table S6; Data sets S6 through S8). We found that of the PULs shown to be differentially expressed in the RNA sequencing, only PUL1, PUL31, and PUL68 were also altered in the OMV fraction (Fig. 5D; Data sets S5 through S8). In addition, PUL17 (host glycans), PUL45 (host glycans), PUL67 (mucin O-glycans), PUL80 (host glycans), and PUL88 (unknown) are altered in the OMV fraction, while PUL10 (unknown), PUL18 (unknown), PUL25 (galactooligosaccharides), PUL30 (mucin), PUL52 (unknown), and PUL80 (host glycans) are changed in the TM fractions, although these PULs were not differentially expressed at the transcriptional level (Fig. 5D; Data set S5 through S8). Previous studies have shown that *Bt* selectively tailors its OMV cargo based on the extracellular nutrient landscape (11). However, our findings are the first to implicate the Dma family in shaping OMV protein cargo through the modulation of PULs in *Bt*.

### *Δdma2* exhibits delayed growth on select host-associated glycans *in vitro*

**Fig 6:**
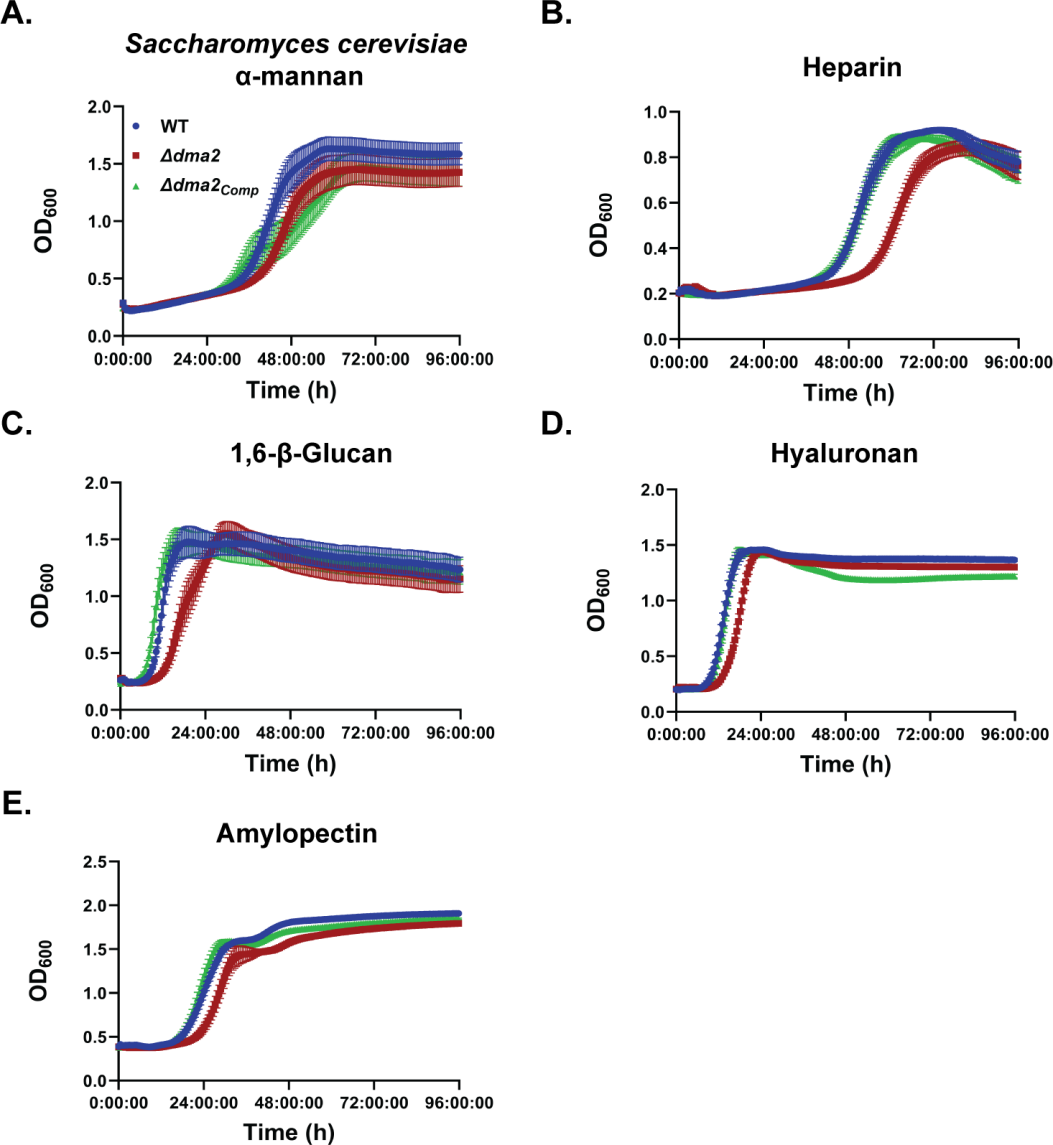
*Δdma2* exhibits delayed growth on select host-derived and microbially derived glycans. Growth curves showing the growth of *Bt* WT, *Δdma2*, and *Δdma2_Comp_* in the presence of minimal media containing (**A**) *Saccharomyces cerevisiae* α-mannan, (**B**) heparin, (**C**) 1,6-β-glucan, (**D**) hyaluronan, and (**E**) amylopectin. Growth curves were generated from the results of at least three independent experiments, each including four technical replicates from each strain. (Supported by Fig. S5). Time points and error bars on the graph represent the mean and standard error of the mean.

Since *Δdma2* represses the activity of many PULs that primarily target host and microbial glycans (Fig. 5D; Tables S5 and S6; Data set S5 through S8), we hypothesized that *Δdma2* would exhibit stunted growth compared to the WT when grown in minimal media supplemented with these types of glycans as a sole carbon source. PUL68 is important for growth in the presence of yeast α-mannan and was the only PUL shown to be significantly repressed in *Δdma2* for each of our comparative analyses (Fig. 5D). Interestingly, we found that *Δdma2* exhibited slower exponential phase growth compared to the WT and complemented strains when grown in the presence of yeast α-mannan, while the lag phase was unaffected (Fig. 6A; Fig. S5A). Together, these findings suggest that when yeast α-mannan is present, *Δdma2* can induce the expression of PUL68 but likely not as efficiently or to the same extent as the WT. Since yeast α-mannans represent a diverse group of polysaccharides, it is possible that growth kinetics would differ in the presence of other α-mannan types (22).

To determine whether the dysregulation of PULs in *Δdma2* impacts their growth in the presence of other carbon sources *in vitro*, we tested a panel of monosaccharides (glucose, fructose, galactose, arabinose, mannose, rhamnose, and xylose), plant polysaccharides (amylopectin, gum arabic, levan, pectin, and rhamnogalacturonan), and host-derived and microbially derived glycans (hyaluronan, heparin, 1,6-β-glucan, and porcine mucin type II/III). When monosaccharides and most plant polysaccharides were the sole carbon source, *Δdma2* grew comparable to the WT (Fig. S6). However, we found that *Δdma2* exhibited delayed growth in the presence of the host-associated glycans, such as heparin, which is present in the intestinal mucosa and secreted by mast cells in the gut; 1,6-β-glucan, which is primarily found in yeast and fungal cell walls; and hyaluronan, which is a component of the extracellular matrix in different mammalian cell types (Fig. 6B through D; Fig. S5B through D) (28–30). On the other hand, amylopectin was the only plant polysaccharide where *Δdma2* exhibited altered growth kinetics, with a longer lag phase but no difference in exponential phase growth rate (Fig. 6E; Fig. S5E). Our findings confirm that *Δdma2* is less able to utilize select carbon sources, primarily host-associated glycans, *in vitro*.

### Dma2 is an important determinant of *in vivo* fitness in *Bt*

**Fig 7:**
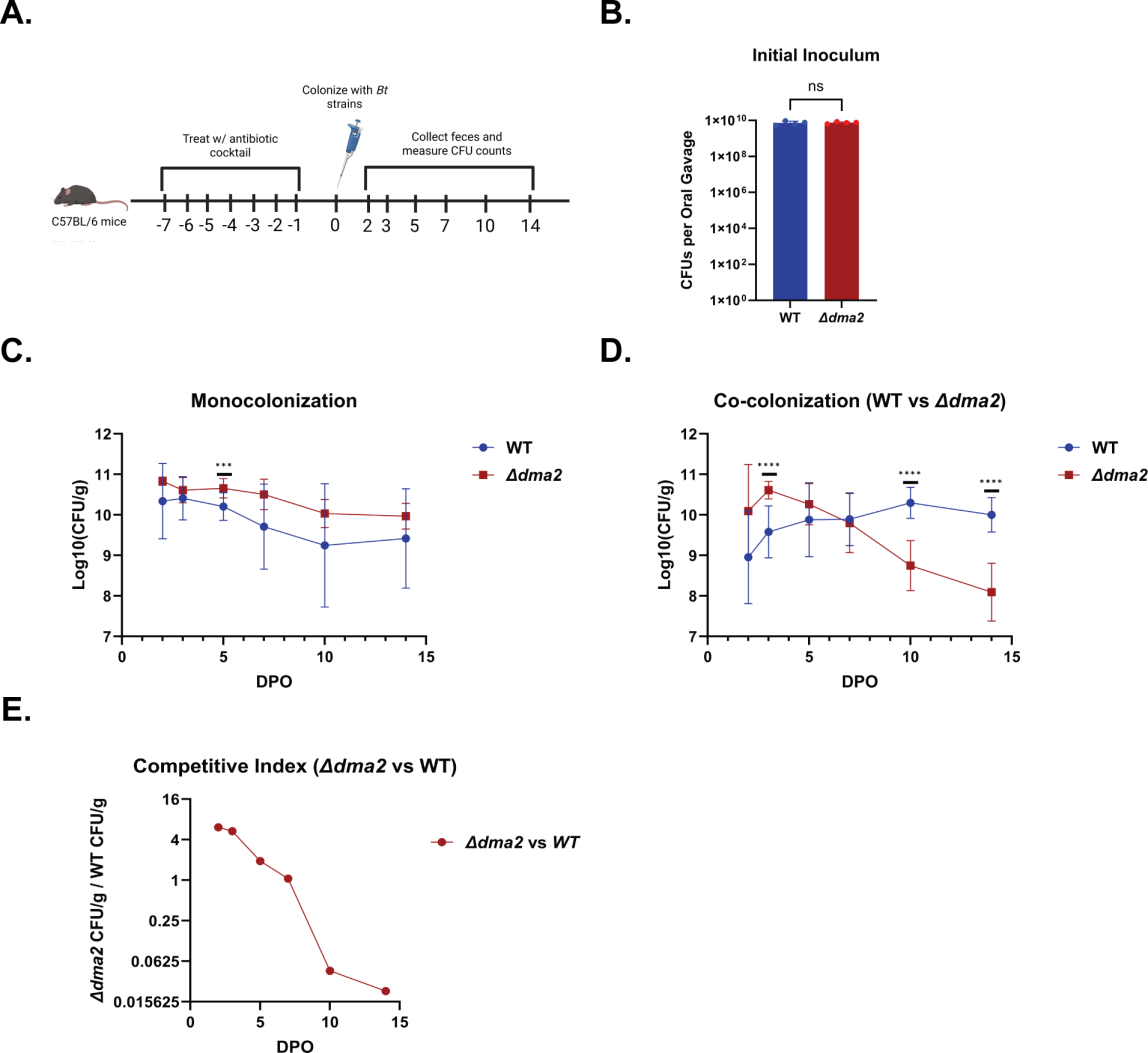
Dma2 is an important colonization factor in *Bt*. (**A**) Schematic outlining *in vivo* colonization of antibiotic-treated mice experiments (Created with BioRender.com). (**B**) Initial inocula for both WT and *Δdma2* show that mice were colonized with equal amounts of each strain from the start. (**C**) Monocolonization studies comparing *Bt* WT and *Δdma2*. This experiment shows that *Bt* WT and *Δdma2* colonize to comparable levels *in vivo*. (**D, E**) Co-colonization experiment comparing *Bt* WT and *Δdma2*. This shows that *Δdma2* is unable to maintain stable colonization when the WT is present. Points on the graph represent the mean and standard deviation of data collected from three independent experiments containing four mice each per condition. Two-tailed unpaired *t*-tests were performed to determine statistical significance. Significance threshold corresponds to: (*) *P*-value ≤ 0.05, (**) *P*-value ≤ 0.01, (***) *P*-value ≤ 0.001, and (****) *P*-value ≤ 0.0001.

PULs that target host-associated glycans are important for *Bt* to stably colonize the human gut (23). Since *Δdma2* displayed delayed *in vitro* growth primarily in the presence of host-associated glycans (Fig. 6A through D), we hypothesized that *Δdma2* may be defective in colonizing *in vivo*. To test this, we treated C57/BL6 mice with an antibiotic cocktail for 7 days prior to colonizing with either *Bt* WT or *Δdma2* by oral gavage and measuring CFUs in feces in intervals for 14 days (Fig. 7A). Mono-colonization experiments revealed that *Δdma2* colonized to the same degree as the WT in our antibiotic-treated mouse model (Fig. 7B and C). On the other hand, co-colonization with the WT and *Δdma2* strains revealed that *Δdma2* initially colonizes at higher levels than the WT, but after 5 days post oral gavage, *Δdma2* rapidly declines in abundance, while the WT remains stable in the population (Fig. 7D and E). To ensure that the fitness defect in *Δdma2* is not due to the overexpression of the genes surrounding *dma2*, we also co-colonized with the WT and *Δbt_1555-dma2,* which possesses *das2* but lacks *dma2* and the rest of the neighboring genes shown previously to be upregulated in *Δdma2*. We confirm that *Δbt_1555-dma2* is still outcompeted by the WT, but unlike *Δdma2*, which initially colonizes better than the WT, *Δbt_1555-dma2* starts to decline in abundance immediately before appearing to stabilize (Fig. S7A and B). Altogether, these findings demonstrate that Dma2 is an important determinant of *in vivo* fitness in *Bt*.

## DISCUSSION

The Dma family is a novel class of anti-sigma factors found solely amongst Bacteroidota (16, 17, 31). To date, members of the Dma family are known to play a role in OMV biogenesis, but we still lack a complete understanding of their role in these microbes. In this study, we showed that deletion of *dma2* in *Bt* results in a significant increase in vesiculation. Dma2-mediated hypervesiculation requires the activity of its cognate sigma factor, Das2. In addition, transcriptome and proteomic analyses show that Dma2 and Das2 are required for proper OMV cargo selection. Finally, we demonstrate through *in vivo* studies that Dma2 is a key determinant of colonization fitness.

Dma1 and Das1 induce OMV production by increasing the expression of *nigD1* (16). On the contrary, we demonstrated that Dma2 and Das2 increase OMV production but do not increase the expression of NigD1 (Table S2; Data set S1). This suggests that Dma1 and Dma2 regulate OMV biogenesis through distinct regulatory cascades. Even so, the role of NigD1 and other NigD-like proteins in *Bt* is currently unknown. Future studies are required to understand the precise mechanism by which Dma2 controls OMV production.

In *Δdma2*, OMV size and structure are not affected (Fig. 2A and B; Fig. S1), but we observed a higher abundance of coccoid cells (Fig. 2C through H). To produce OMVs, cells must properly coordinate the production and trafficking of LPS, other membrane lipids, and OM proteins. This process is likely energetically costly to the bacteria because the secreted cellular contents must be replenished. Our findings could indicate that the induction of OMV biogenesis in *Δdma2* impacts the structure of the cell envelope due to an imbalance between the production and export of OM contents.

We previously showed that when *Bt* is grown in the presence of different polysaccharides, they package their OMVs with enzymes required to degrade them (11). This established a direct link between PUL induction and OMV cargo selection. Interestingly, mucin is a special case where *Bt* induces the requisite PULs, but these components are retained at the cell surface, instead of localizing to OMVs (11). Here, we demonstrate that *Δdma2* primarily alters the expression of various PULs, but many of these transcriptional changes are not necessarily conserved at the protein level (Fig. 5A through D). These findings make Dma2 the first gene shown to impact OMV cargo selection. Most significantly, some of these changes in OMV cargo occur independent of transcriptional regulation, which indicates that additional regulatory features are at play that impact OMV cargo selection in this context. It remains a possibility that Dma2 functions to preclude specific components from host glycan targeting PULs from reaching OMVs. This is physiologically important because it has been postulated that *Bt* aims to avoid degrading host glycans in an uncontrolled fashion because this has been shown to cause inflammation in certain dietary contexts (11, 32).

Comparisons of our transcriptome analyses between *Δdma1* and *Δdma2* revealed that *dma3* is significantly induced in both cases. Very little is known about Dma3, except that it is the most structurally unique member of the Dma family because it is not encoded adjacent to a cognate ECF21 family sigma factor; instead, Dma3 encodes a domain that functions as its cognate sigma factor at the N-terminus of the protein (16). We showed that Dma3 does not impact OMV production in *Δdma1* and *Δdma2* (Fig. S3). However, by comparing transcriptome data sets, we found that *Δdma1* and *Δdma2* both impact the expression of select PULs (Table S3; Data sets S3 and S4); hence, it remains a possibility that Dma3 could play a role in this process. Overall, understanding the role of the Dma family in regulating the PUL repertoire is of significant interest.

In *Bacteroides spp*., PULs are primarily controlled by SusR-like regulators (9, 33), hybrid two-component systems (HTCS) (34–37), and ECF-type sigma/anti-sigma factor systems (23). These systems enable *Bt* to maintain a strict glycan hierarchy where monosaccharides and plant polysaccharides are preferred over host mucosal glycans (23, 38, 39). PULs that target mucin and other host glycans are known to disproportionately be associated with ECF-type sigma/anti-sigma factors. However, to the best of our knowledge, this class of regulators has yet to be shown to be important for maintaining the glycan hierarchy in *Bacteroides spp****.*** (23). Our data demonstrate that Dma2 modulates the activity of PULs; however, it is tempting to speculate that it could play a role in fine-tuning the glycan hierarchy by downregulating select PULs that target host-associated glycans (Fig. 5A, Table S2, S4 and S5; Data sets S1, S2, and S5). Because of this, we hypothesize that Dma2 can sense the presence of a currently unknown glycan at the OM surface and then release Das2 to modulate the activity of PULs from lower priority host glycan targeting PULs.

Our experiments provide preliminary support for this model. In Fig. 6, we demonstrate that *Δdma2* exhibits delayed growth when various host-associated glycans are the sole carbon source. In *Bt*, heparin has been considered a high-priority glycan because PUL85, which targets heparin, is not significantly repressed when monosaccharides are present (39). Our proteomic analyses showed that BT_4659 (SusD-like) and BT_4660 (SusC-like) from PUL85 are repressed in *Δdma2* (Table S6; Data sets S6 and S7), which suggests that Dma2 could be involved in sensing a higher-priority polysaccharide. In tandem, Das2 could function by modulating the activity of lower-priority PULs by inhibiting the activity of their native regulatory systems (23). Future studies should aim to elucidate the substrates that are sensed by Dma2 and decouple whether Das2 functions by directly or indirectly repressing the activity of host glycan-targeting PULs in this context.

Tightly controlling the expression of PULs is key for *Bacteroides spp*. to adapt to and thrive in the human gut (23). Our experiments revealed that the lack of *dma2* drastically impacts the ability of *Bt* to maintain stable colonization when there is competition from other kin bacteria (Fig. 7D and E). We hypothesize that the dysregulation of PULs and OMV cargo selection in *Δdma2* causes the observed fitness defect, but many questions remain. The genes encoded near *dma2* (*bt_1555-bt_1557*) are among the most upregulated genes in *Δdma2*. Our findings demonstrate that these genes are not involved in OMV biogenesis but could be important for the initial colonization fitness of *Δdma2* and potentially the WT (Fig. 4B; Fig. S7). The function of these genes has not been experimentally confirmed; therefore, elucidating the exact function of these genes is required to understand their role *in vivo*.

The Dma2 operon is only encoded in *Bt* and closely related *Bacteroides spp.* (16). This suggests that there is likely a specific context where expressing the *dma2* operon gives these microbes a competitive advantage in the gut. Since many factors can impact colonization fitness, and we only tested mice consuming a standard chow diet, we cannot rule out that the ability of *Δdma2* to colonize *in vivo* may vary depending on the host diet and metabolic state. The gut microbiota also consists of many different bacterial species; so, the lack of colonization fitness observed in *Δdma2* relative to the *Bt* WT begs the question of how this strain would compete in the presence of other *Bacteroides spp.* and commensal microbes (Fig. 7D and E). Future studies will involve determining the exact role of Dma2 and other members of the Dma family within the host.

## MATERIALS AND METHODS

### Bacterial strains and growth conditions

Strains, oligonucleotides, and plasmids are described in Table S1 in the supplemental material. *Escherichia coli* was grown aerobically at 37°C in Luria-Bertani (LB) medium. *Bacteroides* strains were grown in an anaerobic chamber (Coy Laboratories) at 37°C containing an atmosphere of 10% H_2_, 5% CO_2_, 85% N_2_. *Bt* was cultured in Brain Heart Infusion (BHI) medium (Fisher Scientific) supplemented with 5 µg/mL Hemin and 1 µg/mL vitamin K3. When applicable, antibiotics were used as follows: 100 µg/mL ampicillin, 200 µg/mL gentamicin, 25 µg/mL erythromycin, and 10 μg/mL tetracycline. When required, Bacteroides was grown in minimal medium (MM) containing 100 mM KH_2_PO_4_ (pH 7.2), 15 mM NaCl, 8.5 mM (NH4)2SO4, 4 mM L-cysteine, 1.9 mM hematin/200 mM L-histidine (prepared together as a 1,000× solution), 100 mM MgCl2, 1.4 mM FeSO4.7H2O, 50 mM CaCl2, 1 µg/mL vitamin K3, and 5 ng/mL vitamin B12. Carbohydrates used to supplement MM include glucose, fructose, galactose, arabinose, mannose, rhamnose, xylose, amylopectin, gum arabic from Acacia Tree, levan, pectin from citrus peel, rhamnogalacturonan, *Saccharomyces cerevisiae* α-mannan, 1,6-β-glucan, heparin, hyaluronan, porcine mucin type II, porcine mucin type II, porcine mucin type III.

### Genetic manipulation of *Bt*

We employed the pSIE1 vector described in Bencivenga-Barry et al. 2020 to develop constructs for generating clean deletion mutants in *Bt* (40). Briefly, ~750 base pair regions flanking our genes of interest were cloned into pSIE1. Vectors containing flanking regions of target genes were then transformed into *E. coli s17λ-pir* by electroporation. Transformants were identified by selection on LB agar plates containing ampicillin, followed by colony PCR to confirm the presence of the plasmid. Logarithmic to early stationary phase cultures of *E. coli* transformants and *Bt* were mixed (2:1 ratio) to facilitate conjugation of the vector into *Bt*. Transconjugants, containing the vector integrated into the *Bt* genome, were identified by selection on BHI plates containing gentamicin and erythromycin. To delete the gene of interest, *Bt* transconjugants were cultured overnight; to perform counterselection, 5 μL of overnight culture was diluted in 95 μL of BHI media prior to the entire volume being spread on BHI plates containing 125 ng/mL anhydrotetracycline (aTc). Mutants were identified by PCR prior to whole-genome sequencing.

Complementation *of Δdma2* was achieved by cloning *dma2* into the pWW3867 vector backbone under the control of the constitutive RpoD (BT_1311) promoter. This vector was originally designed in the study by Whitaker et al. 2017 (41).

### OMV isolation

OMVs were purified by ultracentrifugation from cell-free culture supernatants according to our previously published methods (10–12, 16). Briefly, 50 mL of *Bt* cultures grown to late stationary phase was centrifuged twice at 6,500 rpm at 4°C for 10 min. Supernatants were then filtered using a 0.22-µm-pore membrane (Millipore) to remove residual cells. The filtrate was subjected to ultracentrifugation at 200,000 × *g* for 2 h (Optima L-100 XP ultracentrifuge; Beckman Coulter). Resulting supernatants were discarded, and the pellets, which contain OMVs, were resuspended in phosphate-buffered saline (PBS). For OMVs, the amount of PBS used for resuspension was based on the measured OD_600_. For example, if the original OD_600_ = 1, then the OMV pellet was resuspended in 100 μL. When performing MS analysis, purified OMV preparations were lyophilized.

### Subcellular fractionation

TM preparations were isolated by cell lysis and ultracentrifugation. Briefly, late stationary phase cultures were harvested by centrifugation at 6,500 rpm at 4°C for 10 min. The pellets were gently resuspended in a mixture of PBS containing complete EDTA-free protease inhibitor mixture (Roche Applied Science). Cells were then lysed using two passes through a cell disruptor at 35 kPa. Next, centrifugation at 8,500 rpm at 4°C for 8 min was performed to remove unbroken cells. TMs were collected by ultracentrifugation at 200,000 × *g* for 1 h at 4°C. Supernatants were discarded, and pellets were resuspended in PBS. For TMs, the amount of PBS used for resuspension was based on the measured OD_600_. If the original OD_600_ = 1, then the TM pellet was resuspended in 1 mL. TM fractions were lyophilized for MS analysis.

### SDS-PAGE analyses

To compare protein profiles from *Bt* WT and *Δdma2* strains, samples, either TM or vesicle fractions, were normalized by OD_600_, and equivalent volumes were loaded onto a 10% Tris-glycine SDS-PAGE gel, followed by Coomassie Blue staining to analyze protein profiles.

Abundance of LPS was measured by adapting the methods from the study of Tsai CM and Frasch CE (42). Briefly, samples were standardized by OD_600_, then diluted in PBS, 1:3 (sample: total volume) for OMVs and 1:5 for TMs, and treated with proteinase K for 4 h at 37°C. Next, 2 μL of proteinase K-digested sample was added to 13 μL of 1× Laemmli buffer and boiled for 3 min prior to loading equal amounts (5–10 μL) onto a 15% SDS-PAGE gel. After running, the gels were fixed overnight in 200 mL of 40% ethanol in 5% acetic acid. Next, the gels were oxidized for 5 min in 100 mL of 0.7% fresh periodic acid in 40% ethanol and 5% acetic acid. Upon completion, gels underwent three washes (15 min each) in milliQ H_2_O. The gels were then stained for 10 min in the dark with 28 mL 0.1M NaOH, 2mL NH_4_OH, 5mL 20% AgNO_3_, and 115 milliQ H_2_O. Gels underwent three additional washes prior to developing in 200 mL H_2_O with 10 mg citric acid and 100 μL formaldehyde.

### Protein quantification

To quantify protein content, we utilized the Bio-Rad *DC* Proteins Assay, which is a colorimetric assay that is like the Lowry assay. Follow the manufacturer’s instructions when performing the assay. At least three biological replicates were performed for each sample in triplicate. The data presented represent the mean and standard error for each sample tested. Two-tailed unpaired *t*-tests were performed to determine significance.

### Growth assay with *Bt* strains

*Bt* WT and *Δdma2* strains were cultured overnight at 37 °C under anaerobic conditions in BHI broth. Overnight cultures were centrifuged at 6,500 rpm for 10 min before being washed with PBS. The cells were then resuspended in MM supplemented with the indicated carbon sources (0.5% wt/vol final concentration) and normalized to OD_600_ =0.1. Growth assays were conducted in sterile, round-bottom 96-well polystyrene microplates. Cultures were incubated anaerobically at 37 °C under static conditions. OD_600_ readings were recorded every 30 min using a Smart Reader 96-T (Accuris Instruments), following 10 s of orbital shaking to ensure homogenization. Each growth condition was tested in technical quadruplicate and independently repeated for at least three biological replicates. Growth curves were determined to be significantly different by measuring (i) the duration of the lag phase, period before bacteria are actively dividing, and (ii) the growth rate during exponential phase (ΔOD_600_/ΔTime (h)), which was calculated by using the OD_600_ at the start of exponential phase growth and the maximum OD_600_ prior to the plateau of bacterial growth when they enter stationary phase. Lag phase duration and exponential phase growth rate were determined for all technical and biological replicates. These data were compiled, and two-tailed unpaired *t*-tests were employed to determine significance. Statistics were provided for growth curves that were found to be significantly altered.

### Cryo-ET sample preparation and imaging

*Bt* cells were grown on BHI-agar plates as described previously. Subsequently, the cells were collected from the plate using a loop and resuspended in 1 mL of 1× PBS to a final OD_600_ of 3.0 for cryo-ET experiments. R2/2 carbon‐coated 200 mesh copper Quantifoil grids (Quantifoil Micro Tools) were glow‐discharged for 30 s. Then, the cells were mixed with a solution of 10‐nm gold beads treated with bovine serum albumin; 4 μL of this mixture was applied to the grids in a Vitrobot chamber (FEI). Subsequently, the extra fluid was blotted off using a Whatman filter paper in the Vitrobot chamber with 100% humidity, and the grids were plunge‐frozen in a cryogen (liquid ethane). Sample imaging was performed at the Advanced Electron Microscopy at the University of Chicago. Cells were imaged using a Titan Krios transmission electron microscope operating at 300 kV and equipped with a BioQuantum K3 imaging filter (Gatan). Data were collected using Tomography 5 software with each tilt series ranging from −60° to 60° in 3° increments with a pixel size of 3.35 Å, an underfocus of 7 μm, and a total dose of 130 e^-^/Å (2). Subsequently, three‐dimensional reconstructions of tilt series and further visualization were performed using the IMOD software package (43).

To determine cell morphology, we measured two perpendicular axes through the center of the cell. Cells where the ratio between these two axes was ~1–1.3 (to allow for inaccuracy in measuring the axes) were classified as coccoid, while those where the ratio was >1.4 were classified as bacilli.

### RNA sequencing sample collection, library preparation, and analysis

RNA was isolated from *Bt* cells according to our methods outlined in the study of Pardue et al. (16). Briefly, WT and *Δdma2* were grown overnight in BHI media before being diluted to the equivalent of OD 0.1 in 10 mL and grown anaerobically for 4 h at 37°C. Four individual overnight and 10 mL culture biological replicates were prepared. Cultures were normalized, and an amount of culture equivalent to an OD_600_ of 4.0 was pelleted for 90s at 8,000 rpm. Pellets were resuspended on ice in 1 mL TRIzol (Invitrogen) with 10 μL of 5 mg/mL glycogen. Samples were flash frozen and stored at −80°C until extraction. Prior to extraction, samples were thawed on ice, then pelleted, and supernatants were treated with chloroform. RNA was extracted from the aqueous phase using the RNeasy minikit (Qiagen, Inc.), and RNA quality was checked by agarose gel electrophoresis and *A*_260_/*A*_280_ measurements. RNA was stored at −80°C with SUPERase-IN RNase inhibitor (Life Technologies) until library preparation.

RNA sequencing prep (RNA-Seq) was performed as previously described (44). Briefly, 400 ng of total RNA from each sample was used for generating cDNA libraries following our RNAtag-Seq protocol. PCR amplified cDNA libraries were sequenced on an Illumina NextSeq500, obtaining a high-sequencing depth (over 7 million reads per sample). RNA-seq data were analyzed using our *in-house* developed analysis pipeline, *Aerobio*. Raw reads are demultiplexed by 5’ and 3’ indices, trimmed to 59 base pairs, and quality filtered (96% sequence quality>Q14). Filtered reads are mapped to the corresponding reference genomes using bowtie2 with the --very-sensitive option (-D 20 –R 3 –N 0 –L 20 –i S, 1, 0.50). Mapped reads are aggregated by feature Count, and differential expression is calculated with DESeq2 (44). In each pair-wise differential expression comparison, significant differential expression is filtered based on two criteria: |log2foldchange| > 1 and adjusted *P*-value (*padj*) <0.05. All differential expression (DE) comparisons are made between the WT and *Δdma*2 mutants under the conditions mentioned above. The reproducibility of the transcriptomic data was confirmed by an overall high Spearman correlation across biological replicates (R > 0.95). BioProject: PRJNA1298834.

### Sample preparation for proteomic analysis

WT, *Δdma2*, and *Δdma2-das2* were grown overnight anaerobically in 3 mL of BHI media prior to being diluted into 50 mL and grown for 20 h. Whole cells, total membranes, and vesicles were collected from each strain. Four individual biological replicates of each fraction were performed for each strain. Samples were lyophilized in preparation for MS analysis.

### Proteomic analysis

Acetone-precipitated protein biological replicates/fractions were solubilized in 4% SDS, 100 mM HEPES by boiling for 10 min at 95 °C, then protein concentrations were assessed using bicinchoninic acid protein assays (Thermo Fisher Scientific). 200 μg of each biological replicate/fraction was prepared for digestion using S-trap mini columns (Protifi, USA) according to the manufacturer’s instructions. Briefly, samples were reduced with 10 mM dithiothreitol for 10 min at 95 °C and then alkylated with 40 mM Iodoacetamide in the dark for 1 h. Samples were acidified to 1.2% phosphoric acid and diluted with seven volumes of S-trap wash buffer (90% methanol, 100 mM Tetraethylammonium bromide, pH 7.1) before being loaded onto S-traps and washed 3 times with 400 μL of S-trap wash buffer. Samples were then digested with 4 μg of Trypsin (a 1:50 protease/protein ratio) in 100 mM Tetraethylammonium bromide overnight at 37 °C before being collected by centrifugation with washes of 100 mM Tetraethylammonium bromide, followed by 0.2% formic acid, and then 0.2% formic acid/50% acetonitrile. Samples were dried down and further cleaned up using C18 Stage (1, 2) tips to ensure the removal of any particulate matter.

C18 cleaned up peptide samples were re-suspended in Buffer A* (2% acetonitrile, 0.1% trifluoroacetic acid in Milli-Q water) and separated using a two-column chromatography set-up on a Dionex Ultimate 3000 UPLC composed of a PepMap100 C18 20 mm × 75 μm trap and a PepMap C18 500 mm × 75 μm analytical column (Thermo Fisher Scientific) coupled to a Orbitrap Fusion™ Lumos Tribrid Mass Spectrometer (Thermo Fisher Scientific) with a FAIMS Pro interface (Thermo Fisher Scientific); 145-minute gradients were run for each sample, with samples loaded onto the trap column with 98% Buffer A (2% acetonitrile, 0.1% formic acid in Milli-Q water) and 2% Buffer B (80% acetonitrile, 0.1% formic acid) with peptides separated by altering the buffer composition from 2% Buffer B to 28% B over 126 min, then from 28% B to 40% B over 9 min, then from 40% B to 80% B over 3 min, the composition was held at 80% B for 2 min, then dropped to 2% B over 2 min, and held at 2% B for another 3 min. A data-dependent stepped FAIMS approach was utilized with two different FAIMS CVs of −45 and −65, as previously described (3). For each FAIMS CV, a single Orbitrap MS scan (500–2,000 m/z, maximal injection time of 50 ms, an AGC of maximum of 4*10^5^ ions and a resolution of 60k) was acquired every 2 s, followed by Orbitrap MS/MS HCD scans of precursors (NCE 30%, maximal injection time of 80 ms, an AGC set to a maximum of 1.25*10^5^ ions and a resolution of 30k).

### Proteomic data analysis

Prior to identification and LFQ analysis, files were separated into individual FAIMS fractions using the FAIMS MzXML Generator (4). Separated FAIMS fractions were searched against the *Bt* VPI-5482 proteome (Uniprot: UP000001414) using MaxQuant (v1.6.17.0) (5), allowing carbamidomethylation of cysteine set as a fixed modification and oxidation of methionine as a variable modification. Searches were performed with Trypsin cleavage specificity, allowing two miscleavage events with a maximum false discovery rate (FDR) of 1.0% set for protein and peptide identifications. The LFQ and “Match Between Run” options were enabled to allow comparison between samples. The resulting data files were processed using Perseus (v1.4.0.6) (6) with missing values imputed based on the total observed protein intensities with a range of 0.3 σ and a downshift of 1.8 σ. Statistical analysis was undertaken in Perseus using two-tailed unpaired *t*-tests. Individual proteins were deemed significantly altered if the “Student’s *t*-test Difference” (equivalent to Log_2_FoldChange) was greater than |±1| and the “-Log Student’s *t*-test *P*-value” was greater than 1.3 (equivalent to *P*-value = 0.05).

### Negative staining and analysis by TEM

For quantitative analyses at the ultrastructural level, 200 mesh formvar/carbon-coated copper grids (Ted Pella Inc., Redding, CA) were coated with 50µg/mL poly-L-lysine (Sigma, St Louis, MO) for 10 min at 37^○^C. Excess fluid was removed, and grids were allowed to air dry. Poly-L-lysine coating allowed for even distribution of material across the grid; 5 μL spots of normalized OMV fractions from our strains were fixed with 1% glutaraldehyde (Ted Pella Inc.) and allowed to absorb onto freshly glow-discharged poly-L-lysine-coated grids for 10 min. Grids were then washed in dH_2_O and stained with 1% aqueous uranyl acetate (Ted Pella Inc.) for 1 min. Excess liquid was gently wicked off, and grids were allowed to air dry. Samples were viewed on a JEOL 1200EX transmission electron microscope (JEOL USA, Peabody, MA) equipped with an AMT 8-megapixel digital camera (Advanced Microscopy Techniques, Woburn, MA). Three biological replicates were prepared for each strain, and each biological replicate was processed in triplicate (three grids per biological replicate for a total of nine grids for each of the three strains tested here). Ten random images were taken at a magnification of 25,000× from various areas of each grid for a total of 90 images per strain. Finally, the total number of OMVs on each grid was manually counted. The gathered data were used to construct violin plots that show the median, interquartile range, and overall data distribution. Two-tailed unpaired *t*-tests were performed to determine statistical significance.

### Competitive colonization of antibiotic-treated mice

All animal experiments were approved by the Washington University Animal Care and Use Committee, and we have complied with all relevant ethical regulations. All mice used were from the inbred C57/BL6 line. Six-week-old animals were used for colonization experiments. Mice were administered an antibiotic cocktail consisting of ampicillin (333.3 mg/mL; 15 μL), neomycin (333.3 mg/mL; 15 μL), metronidazole (10 mg/mL; 100 μL), and vancomycin (166.7 mg/mL; 30 μL), each mouse receiving 160 μL by oral gavage, every 24 h for 7 consecutive days to deplete the normal intestinal flora. Next, mice were given an inoculum of a single *Bt* strain, for monocolonization experiments, or two *Bt* strains, for co-colonization experiments (~10^10^ CFUs/oral gavage total; an aliquot was taken from the input inoculum and plated on BHI agar to count CFUs) for 2 consecutive days. To differentiate our strains, the WT expresses an erythromycin resistance cassette, while the mutants express tetracycline resistance cassettes from the pNBU2 backbone. Fresh fecal pellets were collected 2, 3, 5, 7, 10, and 14 days post-oral gavage and used to quantify CFU/mL to track colonization throughout the duration of the experiment. Four mice were utilized per condition, and each experiment was conducted in triplicate for a total of 12 mice per condition. Competitive index represents the ratio of mutant CFU/g of feces to that of the WT. Two-tailed unpaired *t*-tests were performed to determine statistical significance.

## Data Availability

The mass spectrometry proteomics data has been deposited in the Proteome Xchange Consortium via the PRIDE partner repository (https://www.ebi.ac.uk/pride/) and is accessible with the data set identifier: PXD066605.

## SUPPLEMENTAL MATERIAL

Data Set S1 jb.00442-25-s0001.xlsx RNA sequencing data for Δ*dma2* vs. WT.

Data Set S2 jb.00442-25-s0002.xlsx Polysaccharide utilization loci in *Bacteroides thetaiotaomicron*.

Data Set S3 jb.00442-25-s0003.xlsx Genes upregulated in Δ*dma2* and Δ*dma1*.

Data Set S4 jb.00442-25-s0004.xlsx Genes downregulated in Δ*dma2* and Δ*dma1*.

Data Set S5 jb.00442-25-s0005.xlsx RNA sequencing data for Δ*dma2* vs Δ*dma2-das2*.

Data Set S6 jb.00442-25-s0006.xlsx OMV proteomics data for Δ*dma2* vs Δ*dma2-das2*.

Data Set S7 jb.00442-25-s0007.xlsx TM proteomics data for Δ*dma2* vs. Δ*dma2-das2*.

Data Set S8 jb.00442-25-s0008.xlsx List of polysaccharide utilization loci from Δ*dma2* vs. Δ*dma2-das2* omics.

Supplemental figures jb.00442-25-s0009.pdf Fig. S1 to S7.

Supplemental tables jb.00442-25-s0010.xlsx Tables S1 to S6.

ASM does not own the copyrights to Supplemental Material that may be linked to, or accessed through, an article. The authors have granted ASM a non-exclusive, world-wide license to publish the Supplemental Material files. Please contact the corresponding author directly for reuse.

## References

[1] Sender R, Fuchs S, Milo R. 2016. Revised estimates for the number of human and bacteria cells in the body. PLOS Biol 14:e1002533. doi:10.1371/journal.pbio.1002533 27541692 PMC4991899

[2] Thursby E, Juge N. 2017. Introduction to the human gut microbiota. Biochem J 474:1823–1836. doi:10.1042/BCJ20160510 28512250 PMC5433529

[3] Zafar H, Saier MH. 2021. Gut bacteroides species in health and disease. Gut Microbes 13. doi:10.1080/19490976.2020.1848158 PMC7872030 33535896

[4] Wexler AG, Goodman AL. 2017. An insider’s perspective: bacteroides as a window into the microbiome. Nat Microbiol 2:17026. doi:10.1038/nmicrobiol.2017.26 28440278 PMC5679392

[5] Wexler HM. 2007. Bacteroides: the good, the bad, and the nitty-gritty. Clin Microbiol Rev 20:593–621. doi:10.1128/CMR.00008-07 17934076 PMC2176045

[6] Xu J, Bjursell MK, Himrod J, Deng S, Carmichael LK, Chiang HC, Hooper LV, Gordon JI. 2003. A genomic view of the human-Bacteroides thetaiotaomicron symbiosis. Science 299:2074–2076. doi:10.1126/science.1080029 12663928

[7] Hao Z, Wang X, Yang H, Tu T, Zhang J, Luo H, Huang H, Su X. 2021. PUL-mediated plant cell wall polysaccharide utilization in the gut bacteroidetes. Int J Mol Sci 22:3077. doi:10.3390/ijms22063077 33802923 PMC8002723

[8] Koropatkin NM, Martens EC, Gordon JI, Smith TJ. 2008. Starch catabolism by a prominent human gut symbiont is directed by the recognition of amylose helices. Structure 16:1105–1115. doi:10.1016/j.str.2008.03.017 18611383 PMC2563962

[9] Foley MH, Cockburn DW, Koropatkin NM. 2016. The Sus operon: a model system for starch uptake by the human gut bacteroidetes. Cell Mol Life Sci 73:2603–2617. doi:10.1007/s00018-016-2242-x 27137179 PMC4924478

[10] Elhenawy W, Debelyy MO, Feldman MF. 2014. Preferential packing of acidic glycosidases and proteases into Bacteroides outer membrane vesicles. MBio 5:e00909–14. doi:10.1128/mBio.00909-14 24618254 PMC3952158

[11] Sartorio MG, Pardue EJ, Scott NE, Feldman MF. 2023. Human gut bacteria tailor extracellular vesicle cargo for the breakdown of diet- and host-derived glycans. Proc Natl Acad Sci U S A 120:e2306314120. doi:10.1073/pnas.2306314120 37364113 PMC10319031

[12] Valguarnera E, Scott NE, Azimzadeh P, Feldman MF. 2018. Surface exposure and packing of lipoproteins into outer membrane vesicles are coupled processes in Bacteroides. mSphere 3:e00559-18. doi:10.1128/mSphere.00559-18 30404931 PMC6222051

[13] Sartorio MG, Pardue EJ, Feldman MF, Haurat MF. 2021. Bacterial outer membrane vesicles: from discovery to applications. Annu Rev Microbiol 75:609–630. doi:10.1146/annurev-micro-052821-031444 34351789 PMC8500939

[14] Rakoff-Nahoum S, Coyne MJ, Comstock LE. 2014. An ecological network of polysaccharide utilization among human intestinal symbionts. Curr Biol 24:40–49. doi:10.1016/j.cub.2013.10.077 24332541 PMC3924574

[15] Rakoff-Nahoum S, Foster KR, Comstock LE. 2016. The evolution of cooperation within the gut microbiota. Nature New Biol 533:255–259. doi:10.1038/nature17626 PMC4978124 27111508

[16] Pardue EJ, Sartorio MG, Jana B, Scott NE, Beatty WL, Ortiz-Marquez JC, Van Opijnen T, Hsu F-F, Potter RF, Feldman MF. 2024. Dual membrane-spanning anti-sigma factors regulate vesiculation in Bacteroides thetaiotaomicron. Proc Natl Acad Sci U S A 121:e2321910121. doi:10.1073/pnas.2321910121 38422018 PMC10927553

[17] Ndamukong IC, Gee J, Smith CJ. 2013. The extracytoplasmic function sigma factor EcfO protects Bacteroides fragilis against oxidative stress. J Bacteriol 195:145–155. doi:10.1128/JB.01491-12 23104808 PMC3536166

[18] Staroń A, Sofia HJ, Dietrich S, Ulrich LE, Liesegang H, Mascher T. 2009. The third pillar of bacterial signal transduction: classification of the extracytoplasmic function (ECF) sigma factor protein family. Mol Microbiol 74:557–581. doi:10.1111/j.1365-2958.2009.06870.x 19737356

[19] Varadi M, Anyango S, Deshpande M, Nair S, Natassia C, Yordanova G, Yuan D, Stroe O, Wood G, Laydon A, et al.. 2022. AlphaFold protein structure database: massively expanding the structural coverage of protein-sequence space with high-accuracy models. Nucleic Acids Res 50:D439–D444. doi:10.1093/nar/gkab1061 34791371 PMC8728224

[20] van Kempen M, Kim SS, Tumescheit C, Mirdita M, Lee J, Gilchrist CLM, Söding J, Steinegger M. 2024. Fast and accurate protein structure search with Foldseek. Nat Biotechnol 42:243–246. doi:10.1038/s41587-023-01773-0 37156916 PMC10869269

[21] Mihajlovic J, Bechon N, Ivanova C, Chain F, Almeida A, Langella P, Beloin C, Ghigo J-M. 2019. A putative type V pilus contributes to bacteroides thetaiotaomicron biofilm formation capacity. J Bacteriol 201. doi:10.1128/JB.00650-18 PMC6707919 30833358

[22] Cuskin F, Lowe EC, Temple MJ, Zhu Y, Cameron E, Pudlo NA, Porter NT, Urs K, Thompson AJ, Cartmell A, et al.. 2015. Human gut Bacteroidetes can utilize yeast mannan through a selfish mechanism. Nature 517:165–169. doi:10.1038/nature13995 25567280 PMC4978465

[23] Martens EC, Chiang HC, Gordon JI. 2008. Mucosal glycan foraging enhances fitness and transmission of a saccharolytic human gut bacterial symbiont. Cell Host Microbe 4:447–457. doi:10.1016/j.chom.2008.09.007 18996345 PMC2605320

[24] Taketani M, Donia MS, Jacobson AN, Lambris JD, Fischbach MA. 2015. A phase-variable surface layer from the gut symbiont Bacteroides thetaiotaomicron. mBio 6. doi:10.1128/mBio.01339-15 PMC4611039 26419879

[25] Yaung SJ, Deng L, Li N, Braff JL, Church GM, Bry L, Wang HH, Gerber GK. 2015. Improving microbial fitness in the mammalian gut by in vivo temporal functional metagenomics. Mol Syst Biol 11:788. doi:10.15252/msb.20145866 26148351 PMC4380924

[26] Hsieh SA, Donermeyer DL, Horvath SC, Allen PM. 2021. Phase-variable bacteria simultaneously express multiple capsules. Microbiology (Reading) 167:001066. doi:10.1099/mic.0.001066 34224345 PMC8489884

[27] Porter NT, Hryckowian AJ, Merrill BD, Fuentes JJ, Gardner JO, Glowacki RWP, Singh S, Crawford RD, Snitkin ES, Sonnenburg JL, Martens EC. 2020. Phase-variable capsular polysaccharides and lipoproteins modify bacteriophage susceptibility in Bacteroides thetaiotaomicron. Nat Microbiol 5:1170–1181. doi:10.1038/s41564-020-0746-5 32601452 PMC7482406

[28] Bosi A, Banfi D, Bistoletti M, Moretto P, Moro E, Crema F, Maggi F, Karousou E, Viola M, Passi A, Vigetti D, Giaroni C, Baj A. 2022. Hyaluronan: a neuroimmune modulator in the microbiota-gut axis. Cells 11:126. doi:10.3390/cells11010126 PMC8750446 35011688

[29] Cartmell A, Lowe EC, Baslé A, Firbank SJ, Ndeh DA, Murray H, Terrapon N, Lombard V, Henrissat B, Turnbull JE, Czjzek M, Gilbert HJ, Bolam DN. 2017. How members of the human gut microbiota overcome the sulfation problem posed by glycosaminoglycans. Proc Natl Acad Sci U S A 114:7037–7042. doi:10.1073/pnas.1704367114 28630303 PMC5502631

[30] Temple MJ, Cuskin F, Baslé A, Hickey N, Speciale G, Williams SJ, Gilbert HJ, Lowe EC. 2017. A Bacteroidetes locus dedicated to fungal 1,6-β-glucan degradation: unique substrate conformation drives specificity of the key endo-1,6-β-glucanase. J Biol Chem 292:10639–10650. doi:10.1074/jbc.M117.787606 28461332 PMC5481569

[31] Evans JC, McEneany VL, Coyne MJ, Caldwell EP, Sheahan ML, Von SS, Coyne EM, Tweten RK, Comstock LE. 2022. A proteolytically activated antimicrobial toxin encoded on a mobile plasmid of Bacteroidales induces a protective response. Nat Commun 13:4258. doi:10.1038/s41467-022-31925-w 35871068 PMC9308784

[32] Desai MS, Seekatz AM, Koropatkin NM, Kamada N, Hickey CA, Wolter M, Pudlo NA, Kitamoto S, Terrapon N, Muller A, Young VB, Henrissat B, Wilmes P, Stappenbeck TS, Núñez G, Martens EC. 2016. A dietary fiber-deprived gut microbiota degrades the colonic mucus barrier and enhances pathogen susceptibility. Cell 167:1339–1353. doi:10.1016/j.cell.2016.10.043 27863247 PMC5131798

[33] Cho KH, Cho D, Wang G-R, Salyers AA. 2001. New regulatory gene that contributes to control of Bacteroides thetaiotaomicron starch utilization genes. J Bacteriol 183:7198–7205. doi:10.1128/JB.183.24.7198-7205.2001 11717279 PMC95569

[34] Gao R, Wu T, Stock AM. 2024. A conserved inhibitory interdomain interaction regulates DNA-binding activities of hybrid two-component systems in Bacteroides. mBio 15:e0122024. doi:10.1128/mbio.01220-24 38842315 PMC11253607

[35] Lee J-H, Kwon S-J, Han J-Y, Cho S-H, Cho Y-J, Park J-H. 2022. A mucin-responsive hybrid two-component system controls Bacteroides thetaiotaomicron colonization and gut homeostasis. J Microbiol 60:215–223. doi:10.1007/s12275-022-1649-3 35102527

[36] Lynch JB, Sonnenburg JL. 2012. Prioritization of a plant polysaccharide over a mucus carbohydrate is enforced by a Bacteroides hybrid two-component system. Mol Microbiol 85:478–491. doi:10.1111/j.1365-2958.2012.08123.x 22686399 PMC3404733

[37] Schwalm ND 3rd, Townsend GE 2nd, Groisman EA. 2017. Prioritization of polysaccharide utilization and control of regulator activation in Bacteroides thetaiotaomicron. Mol Microbiol 104:32–45. doi:10.1111/mmi.13609 28009067

[38] Pudlo NA, Urs K, Kumar SS, German JB, Mills DA, Martens EC. 2015. Symbiotic human gut bacteria with variable metabolic priorities for host mucosal glycans. mBio 6:e01282-15. doi:10.1128/mBio.01282-15 26556271 PMC4659458

[39] Rogers TE, Pudlo NA, Koropatkin NM, Bell JSK, Moya Balasch M, Jasker K, Martens EC. 2013. Dynamic responses of Bacteroides thetaiotaomicron during growth on glycan mixtures. Mol Microbiol 88:876–890. doi:10.1111/mmi.12228 23646867 PMC3700664

[40] Bencivenga-Barry NA, Lim B, Herrera CM, Trent MS, Goodman AL. 2020. Genetic manipulation of wild human gut bacteroides. J Bacteriol 202:e00544–19. doi: 10.1128/JB.00544-19 31712278 10.1128/JB.00544-19 PMC6964735

[41] Whitaker WR, Shepherd ES, Sonnenburg JL. 2017. Tunable expression tools enable single-cell strain distinction in the gut microbiome. Cell 169:538–546. doi:10.1016/j.cell.2017.03.041 28431251 PMC5576361

[42] Tsai C-M, Frasch CE. 1982. A sensitive silver stain for detecting lipopolysaccharides in polyacrylamide gels. Anal Biochem 119:115–119. doi:10.1016/0003-2697(82)90673-x 6176137

[43] Kremer JR, Mastronarde DN, McIntosh JR. 1996. Computer visualization of three-dimensional image data using IMOD. J Struct Biol 116:71–76. doi:10.1006/jsbi.1996.0013 8742726

[44] Zhu Z, Surujon D, Ortiz-Marquez JC, Huo W, Isberg RR, Bento J, van Opijnen T. 2020. Entropy of a bacterial stress response is a generalizable predictor for fitness and antibiotic sensitivity. Nat Commun 11:4365. doi:10.1038/s41467-020-18134-z 32868761 PMC7458919

