# Supplementary material for: Dual membrane-spanning anti-sigma 2 controls OMV biogenesis and colonization fitness in Bacteroides thetaiotaomicron
Source: J Bacteriol. 2026 Mar 5;208(4):e00442-25. doi: 10.1128/jb.00442-25 (PMC13088901; doi:10.1128/jb.00442-25)
Supplement: Supplemental figures — Fig. S1 to S7. [file jb.00442-25-s0009.pdf]

Supplemental Information for:

Dual Membrane-spanning Anti-Sigma 2 Controls OMV biogenesis and Colonization Fitness in *Bacteroides thetaiotaomicron*

Evan J. Pardue[1], Tengfei Zhong[2], Nichollas E. Scott[3], Biswanath Jana[1], Wandy Beatty[1], Juan C. Ortiz-Marquez[4], Mohammed Kaplan[2], Clay Jackson-Litteken[5], Mario F. Feldman[1#]

[1]Department of Molecular Microbiology, Washington University School of Medicine, Saint Louis, Missouri, USA

[2]Department of Microbiology, University of Chicago, Chicago, Illinois, USA

[3]Department of Microbiology and Immunology, The Peter Doherty Institute for Infection and Immunity, University of Melbourne, Parkville, VIC 3000, Australia

[4]Biology Department, Boston College, Chestnut Hill, Massachusetts, USA

[5]Department of Microbiology and Immunology, University of Arkansas for Medical Sciences, Little Rock, Arkansas, USA

#Address correspondence to Mario F. Feldman, mariofeldman@wustl.edu.

Author order was determined based on individual contribution.

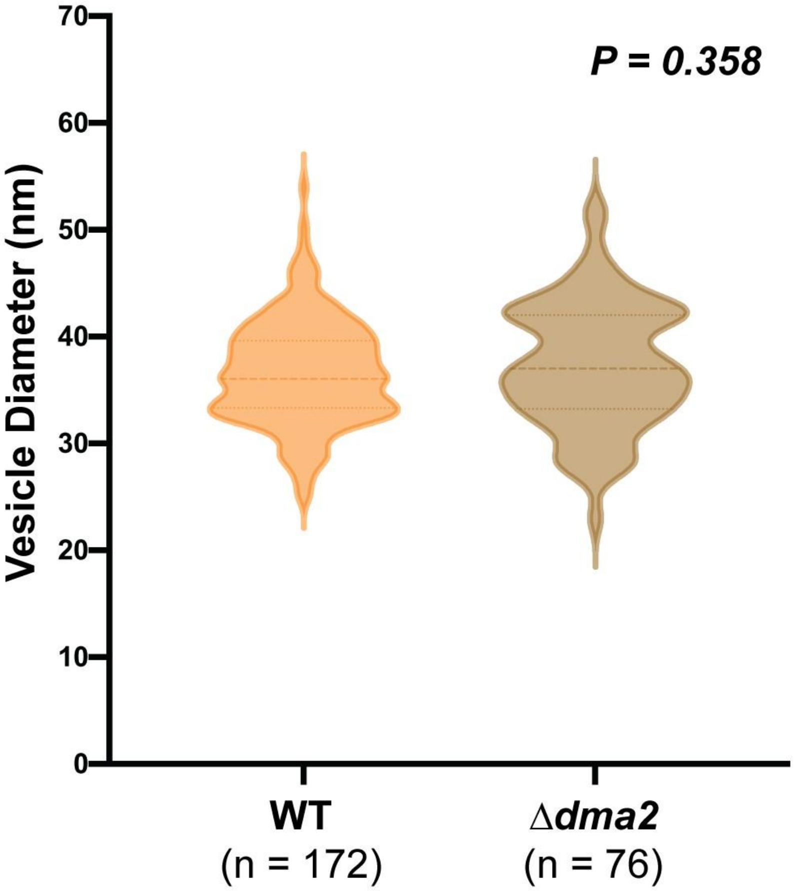

Supplemental Figure 1: OMV diameter distribution in WT and *Δdma2* cells. A violin plot showing the outer membrane vesicles diameters in both WT and *Δdma2* cells. The number of vesicles (n) examined for each sample is indicated on the X-axis.

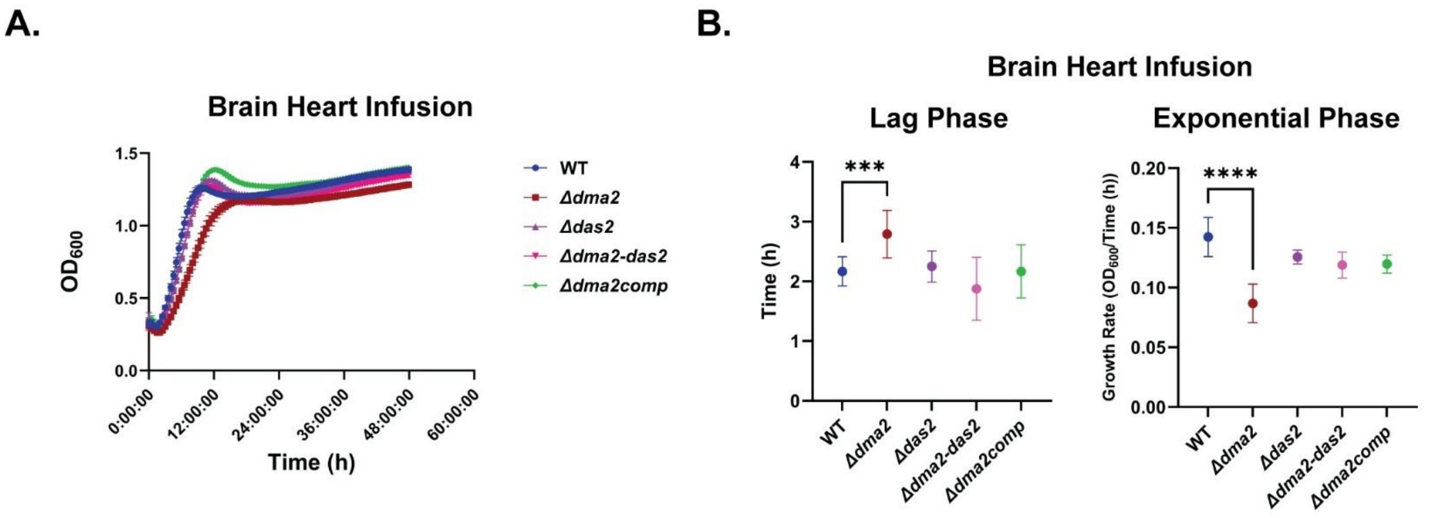

Supplemental Figure 2: Mutation of *das2* does not impact growth *in vitro*. (A) Growth curves showing the growth of *Bt* WT, *Δdma2*, *Δdas2*, *Δdma2-das2*, and $\Delta dma2_{Comp}$ in supplemented BHI broth. Growth curves presented represent the average and standard error of mean from the results of at least three independent biological replicates each including four technical replicates from each strain. Timepoints and error bars represent the mean and standard error of mean. (B) Lag phase duration (*left*) and exponential phase growth rate (*right*) were determined for all technical and biological replicates. These data were compiled, and two-tailed unpaired T-tests were employed to determine significance. Significance threshold corresponds to: (*) p-value ≤ 0.05, (**) p-value ≤ 0.01, (***) p-value ≤ 0.001, (****) p-value ≤ 0.0001.

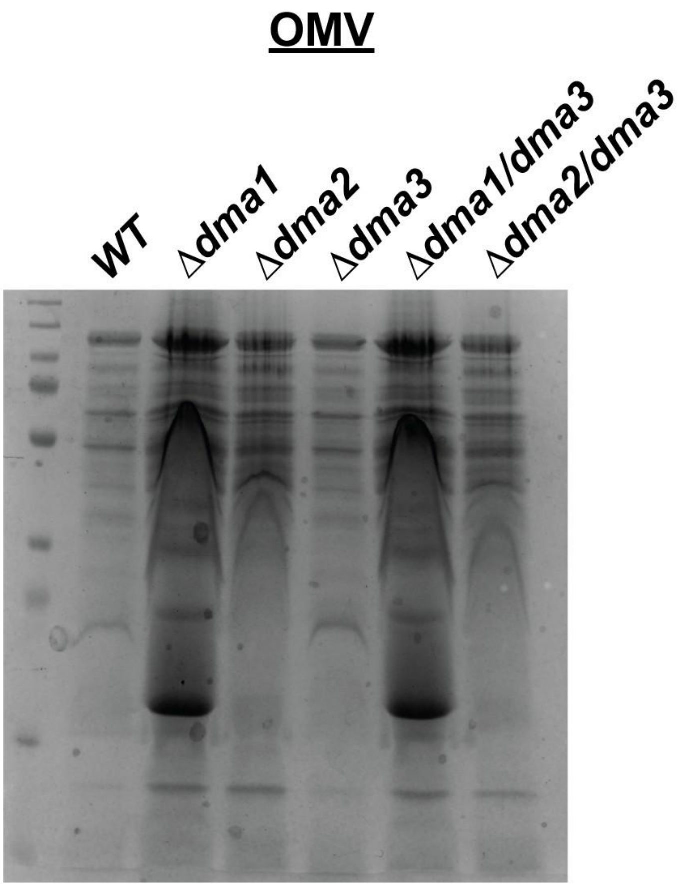

Supplemental Figure 3: Dma3 does not promote OMV biogenesis in *Δdma1* and *Δdma2.* Coomassie Blue stain of OMV fractions isolated from *Bt* WT, *Δdma1, Δdma2*, and the corresponding strains containing deletion in *dma3*. Samples were normalized by OD_{600} and analyzed by 10% SDS-PAGE. This shows that mutation of *dma3* does not impact the electrophoretic profile of the OMV fraction in any of the genetic backgrounds tested.

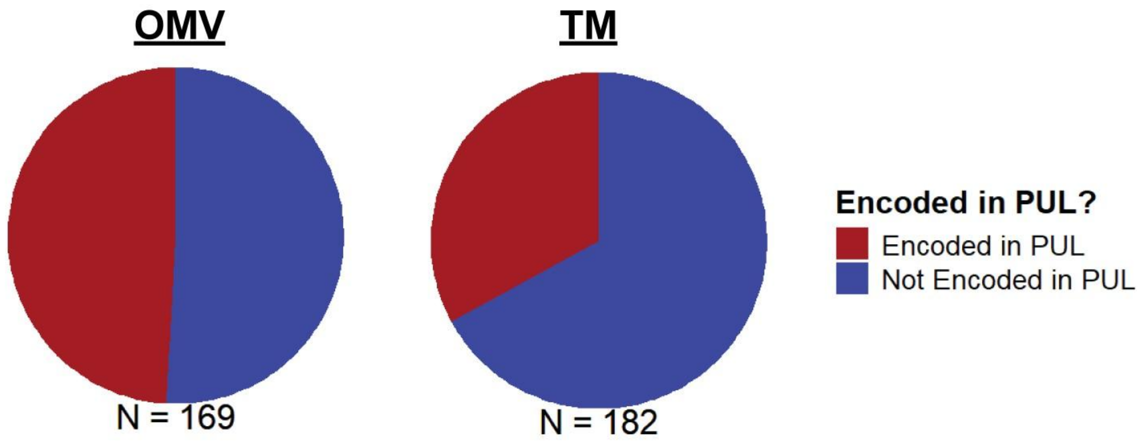

Supplemental Figure 4: PUL encoded proteins are the main group of proteins altered in the OMV fraction of *Δdma2*. Pi charts corresponding to the proportion of significantly altered proteins from the *Bt Δdma2* and *Δdma2-das2* proteomics encoded in PULs **(Dataset S6, S7).**

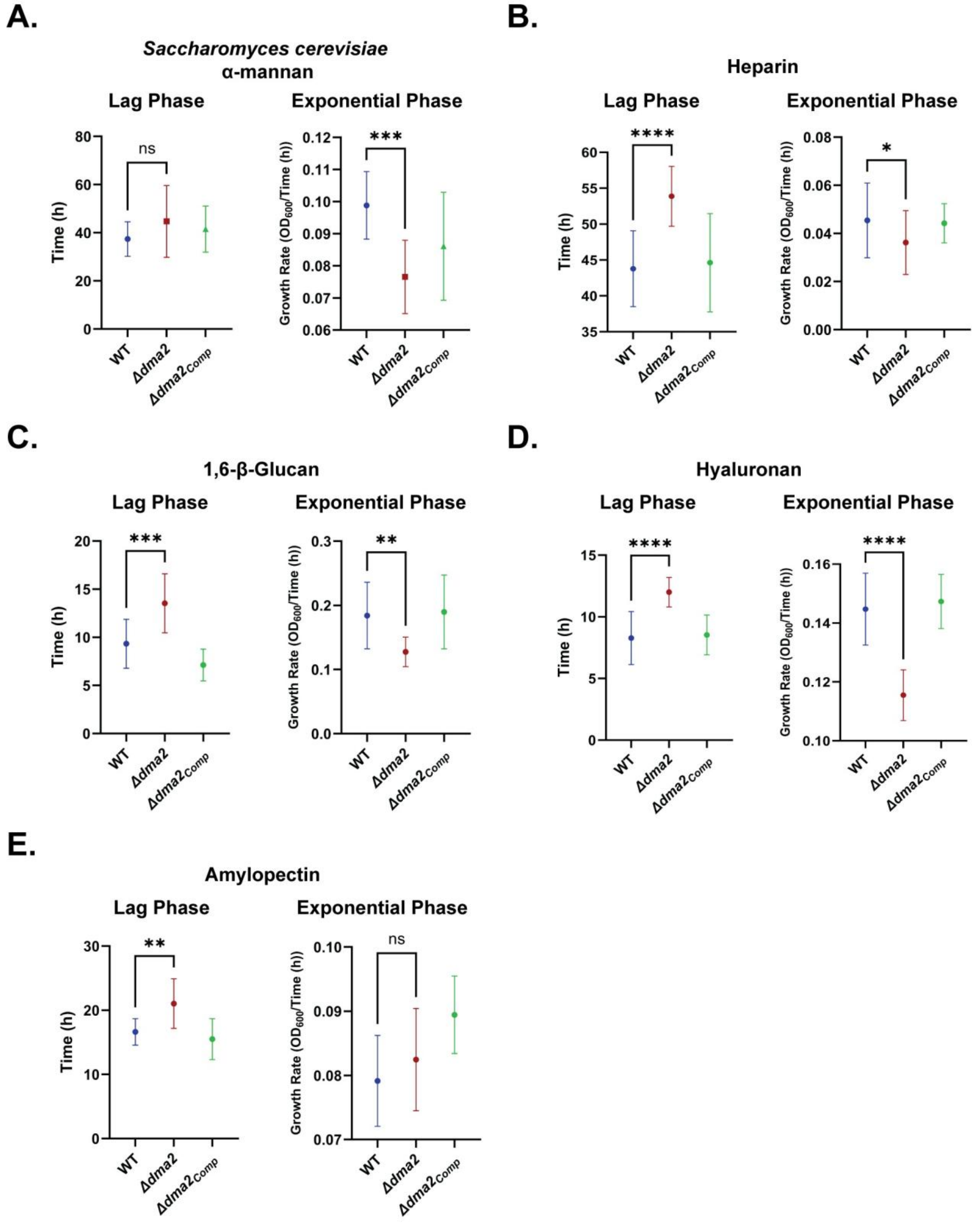

Supplemental Figure 5: Lag phase duration and exponential phase growth rate of *Δdma2* grown in the presence of different glycans. Lag phase duration and exponential phase growth rates were calculated for growth curves of *Bt* WT, *Δdma2*, and *Δdma2*$_{Comp}$ in the presence of (A) *Saccharomyces cerevisiae* α-mannan, (B) Heparin, (C) 1,6-β-Glucan, (D) Hyaluronan, and (E) Amylopectin. Lag phase duration and exponential phase growth rate were determined for all technical and biological replicates.

These data were compiled, and two-tailed unpaired T-tests were employed to determine significance. Significance threshold corresponds to: (*) p-value ≤ 0.05, (**) p-value ≤ 0.01, (***) p-value ≤ 0.001, (****) p-value ≤ 0.0001.

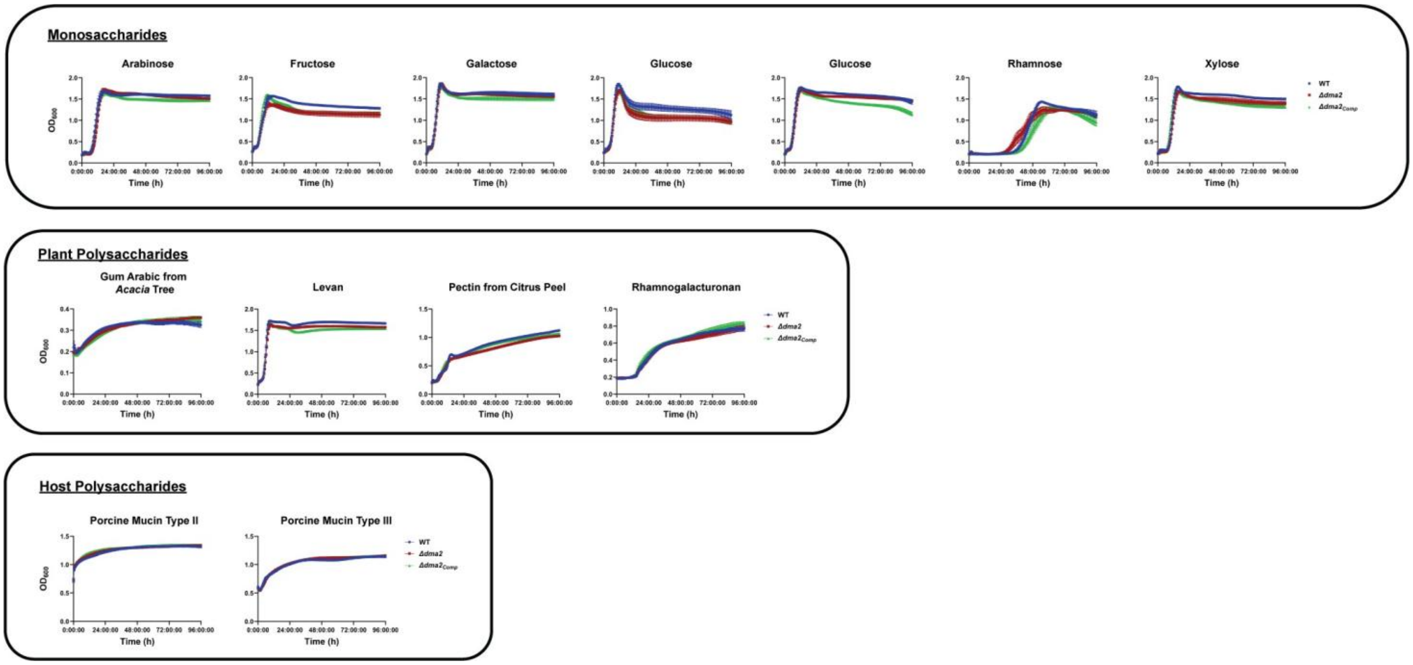

Supplemental Figure 6: Growth of *Δdma2* in the presence of various carbon sources. Growth curves showing the growth of *Bt* WT, *Δdma2*, and *Δdma2$_{Comp}$* in the presence of minimal media containing various monosaccharides and polysaccharides. Growth curves were generated from the results of three independent experiments each including four technical replicates from each strain. Timepoints and error bars on the graph represent the mean and standard error of mean.

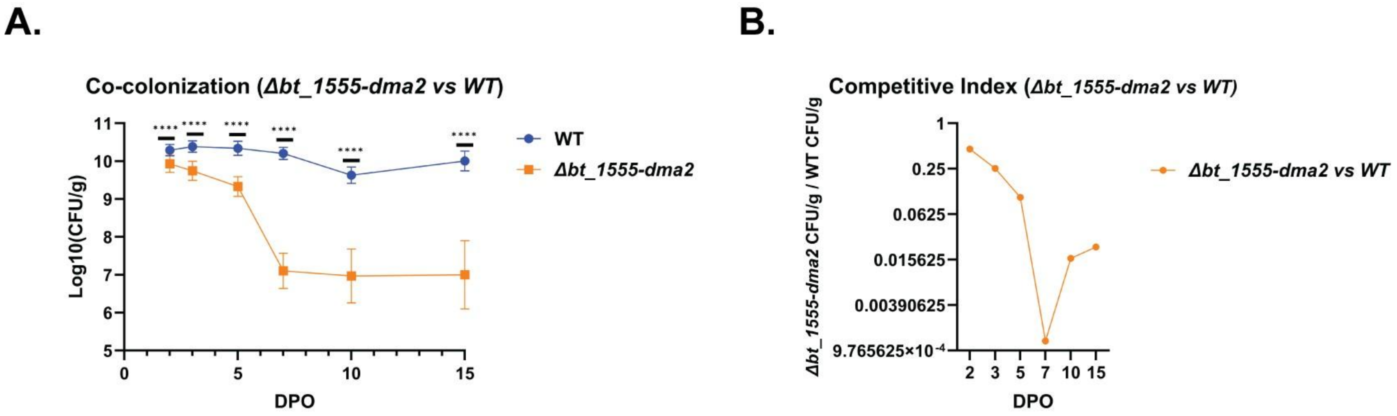

Supplemental Figure 7: Fitness defect *in vivo* is not due to the overexpression of *dma2* operon in *Δdma2*. (A, B) Co-colonization experiment comparing *Bt* WT and *Δ1555-dma2*. This shows that *Δ1555-dma2* experiences and immediate decrease in abundance when the WT is present. This phenotype is similar the that observed in *Δdma2*, which indicates that the overexpression of the downstream genes in the *dma2* operon is not what causes the decline in colonization fitness observed in *Δdma2*. Points on the graph represent the mean and standard deviation of data collected from three independent experiments containing four mice each per condition. Two-tailed unpaired t-tests were performed to determine statistical significance. Significance threshold corresponds to: (*) p-value = 0.05, (**) p-value = 0.01, (***) p-value = 0.001, (****) p-value = 0.0001.

<u>Supplemental Datasets</u>

Supplemental Dataset 1: RNA Sequencing- *Δdma2* vs *WT*

Supplemental Dataset 2: Polysaccharide Utilization Loci in *Bacteroides thetaiotaomicron*

Supplemental Dataset 3: Genes Upregulated in *Δdma2* and *Δdma1*

Supplemental Dataset 4: Genes Downregulated in *Δdma2* and *Δdma1*

Supplemental Dataset 5: RNA Sequencing- *Δdma2* vs *Δdma2-das2*

Supplemental Dataset 6: OMV Proteomics- *Δdma2* vs *Δdma2-das2*

Supplemental Dataset 7: TM Proteomics- *Δdma2* vs *Δdma2-das2*

Supplemental Dataset 8: List of Polysaccharide Utilization Loci from *Δdma2* vs *Δdma2-das2* omics
